# VISUAL-CC system uncovers the role of GSK3 as an orchestrator of vascular cell type ratio in plants

**DOI:** 10.1038/s42003-020-0907-3

**Published:** 2020-04-22

**Authors:** Takayuki Tamaki, Satoyo Oya, Makiko Naito, Yasuko Ozawa, Tomoyuki Furuya, Masato Saito, Mayuko Sato, Mayumi Wakazaki, Kiminori Toyooka, Hiroo Fukuda, Ykä Helariutta, Yuki Kondo

**Affiliations:** 1 Department of Biological Sciences, Graduate School of Science, The University of Tokyo, Tokyo, 113-0033 Japan; 2 Department of Biology, Graduate School of Science, Kobe University, Kobe, 657-8501 Japan; 3 RIKEN Centre for Sustainable Resource Science, Yokohama, 230-0045 Japan; 4 Institute of Biotechnology/Department of Biological and Environmental Sciences, University of Helsinki, FIN-00014 Helsinki, Finland; 5 The Sainsbury Laboratory, University of Cambridge, Cambridge, CB2 1LR UK

**Keywords:** Plant stem cell, Differentiation

## Abstract

The phloem transports photosynthetic assimilates and signalling molecules. It mainly consists of sieve elements (SEs), which act as “highways” for transport, and companion cells (CCs), which serve as “gates” to load/unload cargos. Though SEs and CCs function together, it remains unknown what determines the ratio of SE/CC in the phloem. Here we develop a new culture system for CC differentiation in *Arabidopsis* named VISUAL-CC, which almost mimics the process of the SE–CC complex formation. Comparative expression analysis in VISUAL-CC reveals that SE and CC differentiation tends to show negative correlation, while total phloem differentiation is unchanged. This varying SE/CC ratio is largely dependent on GSK3 kinase activity. Indeed, *gsk3* hextuple mutants possess many more SEs and fewer CCs, whereas *gsk3* gain-of-function mutants partially increase the CC number. Taken together, GSK3 activity appears to function as a cell-fate switch in the phloem, thereby balancing the SE/CC ratio.

## Abstract (web summary)

Tamaki et al. develop VISUAL-CC to study SE–CC (sieve elements–companion cells) complex formation. They show that the balance in the SE/CC ratio is dependent on GSK3 activity using different genetic backgrounds. Their work provides insights on the role of GSK3 as a cell-fate switch in the phloem.

## Introduction

Multicellular organisms possess a variety of functional cells with a proper ratio for their life maintenance. In plants, the phloem tissue is composed of two major cell types: sieve elements (SEs) as conductive tubes and phloem companion cells (CCs) as helper cells for phloem transport. Phloem CCs function to support neighboring SEs through connected plasmodesmata. Although they function together with each other to ensure phloem transport, it has long been a deep mystery how the ratio of SE/CC is strictly controlled in the phloem. Recent technical advances enabled to identify various regulators that control SE differentiation^1^. In contrast to SEs, understanding of the molecular mechanism underlying CC differentiation remains a long-standing challenge.

Vascular cell induction culture system using *Arabidopsis* leaves (VISUAL) is a culture system that can artificially mimic plant vascular development^2^. In the VISUAL system, mesophyll cells are reprogrammed into vascular stem cells, and then differentiated into xylem vessel elements or phloem SEs within a couple of days. VISUAL enables the molecular genetic studies of vascular development, leading to the in-depth understanding of the regulatory network, especially for phloem SE differentiation. Even in VISUAL, differentiation into phloem CCs rarely occurs^2^, which makes it difficult to study CC development in detail.

In this study, we develop a new culture system for inducing CC-like cell differentiation named VISUAL-CC by modifying the conventional VISUAL method. Based on comprehensive gene expression analysis in VISUAL-CC, here we reveal that GLYCOGEN SYNTHASE KINASE 3 (GSK3) activity plays an important role in determining the SE/CC ratio. In vivo genetic analyses confirm the importance of GSK3 probably as a cell-fate switch in phloem development.

## Results

### VISUAL-CC is a culture system for inducing CC-like cells

**Fig. 1:**
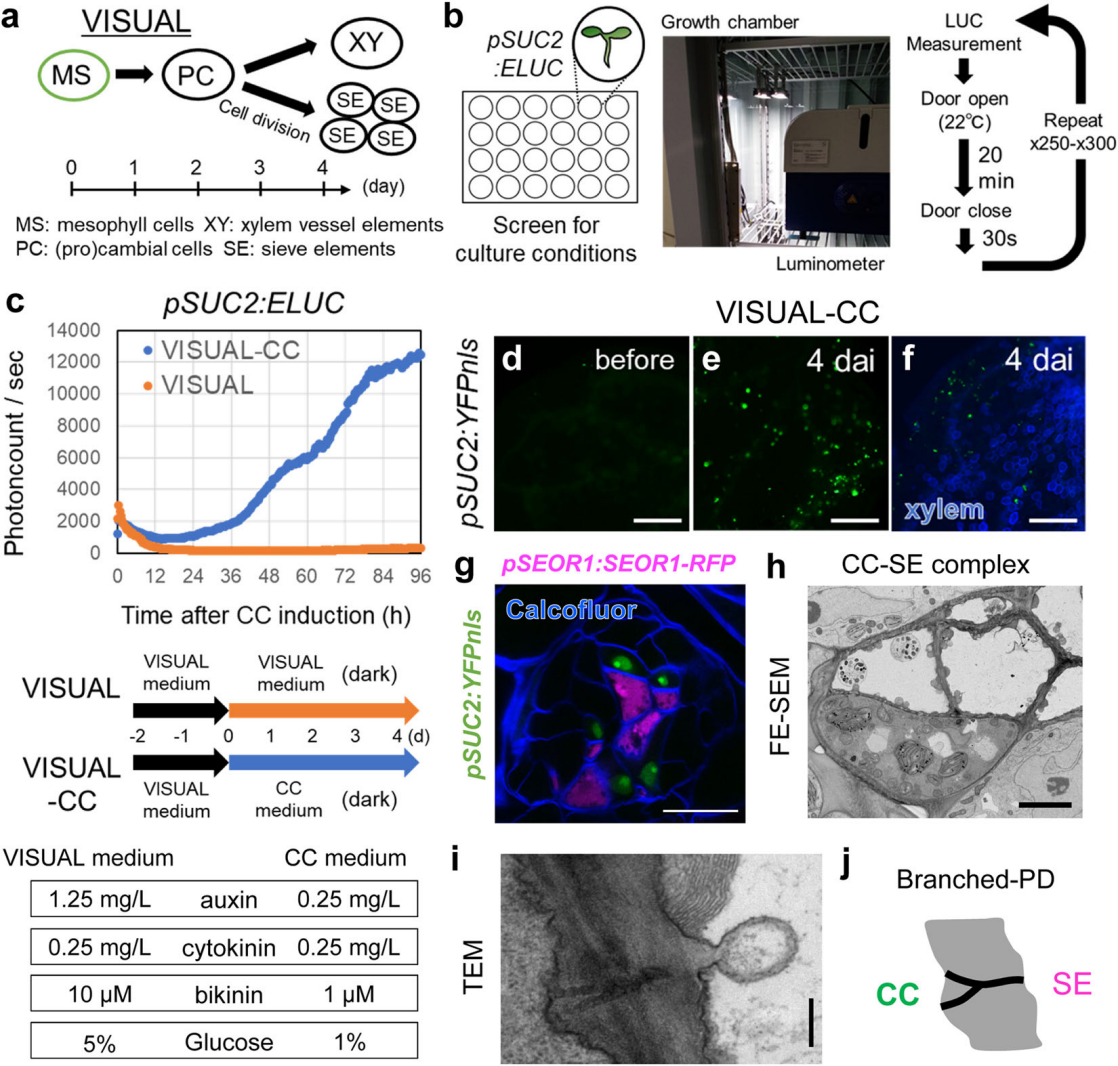
VISUAL-CC is a new method for inducing SE–CC complexes. **a** The process of vascular cell differentiation in conventional VISUAL system. **b** Schematic of the screening system. LUC activity of *pSUC2:ELUC* was monitored every 20 min during culture under various conditions. **c** Time course of *pSUC2:ELUC* intensities during culture in conventional VISUAL and VISUAL-CC. The vertical axis indicates photon counts per second detected by the luminometer. The lower panel illustrates culture conditions and medium composition during VISUAL and VISUAL-CC culture; see Materials for a detailed protocol. **d**, **e** *pSUC2:YFPnls* expression before (**d**) and after 4 days of VISUAL-CC treatment (**e**) in cotyledons. **f** Simultaneous observation of *pSUC2:YFPnls* expression and xylem cells after VISUAL-CC treatment. Xylem cells were detected as UV autofluorescent signal (blue). **g** Expression patterns of *pSEOR1:SEOR1-RFP* (magenta) and *pSUC2:YFPnls* (green) in VISUAL-CC. Cell walls were stained with calcofluor white (blue). **h** FE-SEM image of ectopic CC-like cells (high electron density) and SEs (low electron density) induced by VISUAL-CC. **i** TEM image of branched plasmodesmata between an ectopic CC-like cell and a SE. **j** Schematic of branched plasmodesmata. Scale bars: 500 μm (**d**–**g**); 5 μm (**h**); 200 nm (**i**).

The conventional VISUAL system can induce ectopic xylem (XY) or phloem SEs via the stage of vascular stem cell (Fig. 1a). Toward the understating of CC differentiation, we modified the VISUAL based on a luciferase-based screen with *SUCROSE-PROTON SYMPORTER 2* (*SUC2*) *pro:ELUC*, a specific CC marker^3^ (Fig. 1b). In this screen process, vascular stem cells were induced by the conventional VISUAL method in advance, and subsequently were exposed to a variety of culture media. After a series of screens with different media, we could induce *pSUC2:ELUC* activity (Fig. 1c) and ectopic expression of the *pSUC2:YFPnls* marker in cotyledons within 4 days using CC medium (Fig. 1d–f). Hereafter, we refer to this culture system as “VISUAL-CC”. To further investigate the spatial pattern, a dual phloem marker line expressing *pSUC2:YFPnls* and *SIEVE-ELEMENT-OCCLUSION-RELATED 1* (*SEOR1*) *pro:SEOR1-RFP*^5^, a specific SE marker was established. Detailed observation of the dual phloem marker line by confocal microscopy after tissue-clearing treatment (ClearSee)^4^ revealed that CCs expressing *pSUC2:YFPnls* (green) are detected only in dividing cell clusters and are always limited to the cells adjacent to SEs expressing *pSEOR1:SEOR1-RFP*^5^ (magenta) (Fig. 1g). Thus, CC and SE markers appeared next to each other after several rounds of cell division (Fig. 1g). Observations using a field-emission scanning electron microscope (FE-SEM) or transmission electron microscopy (TEM) consistently visualized CC-like cells with dense cytoplasm adjacent to SEs with brighter cytoplasm (Fig. 1h). These cells showed minor vacuolation and developed the branched plasmodesmata typically seen in SE–CC complexes in vivo (Fig. 1h–j). In VISUAL, SMXL4 and SMXL5 are known as important regulators for early phloem SE development (Supplementary Fig. 1a)^6,7^. In VISUAL-CC, the double mutant *smxl4 smxl5* significantly suppressed CC-like cell differentiation (Supplementary Fig. 1b), suggesting that SE and CC differentiation partially shares a common developmental process from vascular stem cells. Taken together, these results suggest that VISUAL-CC can mimic the SE–CC complex formation.

### VISUAL-CC can induce known CC-related gene expression

**Fig. 2:**
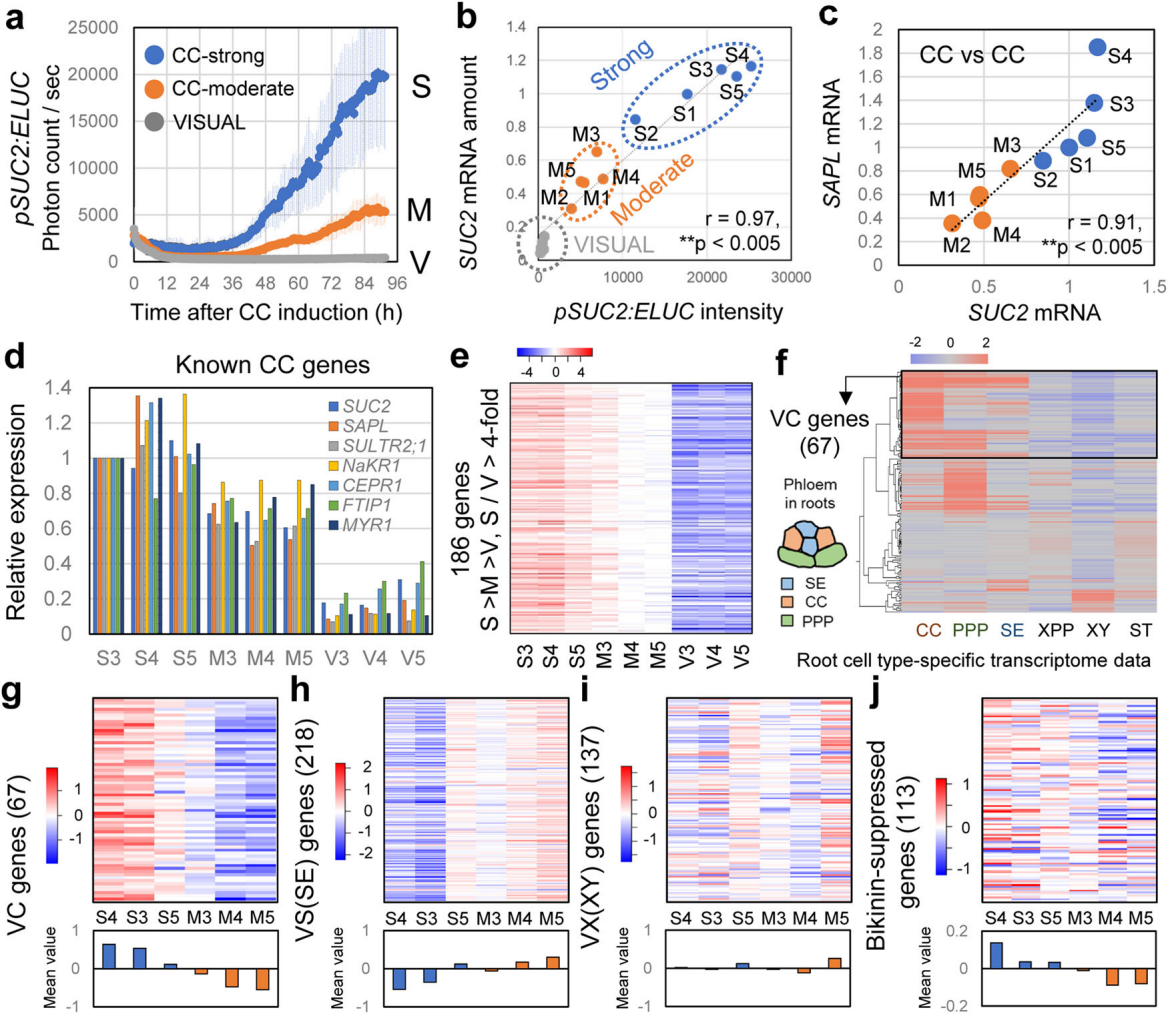
Transcriptome analysis in VISUAL-CC. **a** Time course of *pSUC2:ELUC* signal intensities for each category: CC-strong (S), CC-moderate (M), and VISUAL (V) (*n* = 5). Error bars indicate SD. **b** Correlation between *pSUC2:ELUC* signals and relative *SUC2* mRNA expression levels for each sample. **c** Correlation of expression levels between *SUC2* and *SAPL* in S and M samples. **d** Expression levels of known CC-related genes in each sample. Relative expression levels were calculated when expression in S3 was set to 1. **e** Heat map of expression levels of VISUAL-CC-inducible genes in each sample. Expression data were normalized against the median value and are presented according to the color scale (log2) above the chart. **f** Cluster analysis of VISUAL-CC-inducible genes based on a previous root cell-type-specific transcriptome dataset^14^. A schematic of the primary phloem tissue patterning found in roots is shown on the left-hand side of the chart. **g**–**i** Heat map of expression levels of VC (67 genes, **g**), VS (218 genes, **h**), and VS (137 genes, **i**) in S and M samples. The lower panel indicates the mean value for each sample. **j** Heat map of expression levels of 113 bikinin-suppressed genes in S and M samples.

To validate the promoter-based assay, we compared *SUC2* mRNA accumulation with the promoter:LUC activity in the same sample (Fig. 2a, b). qRT-PCR analyses of VISUAL-CC samples and samples cultured in the conventional VISUAL medium (VISUAL, V) as negative controls revealed a strong correlation between promoter activity and mRNA level of *SUC2* (Fig. 2b; *r* = 0.97, *P* < 0.005) (Fig. 2b). We used these data for classification of VISUAL-CC samples into strong LUC activity (CC-strong, S) and moderate LUC activity (CC-moderate, M), according to their *SUC2* levels (Fig. 2a, b; Supplementary Fig. 2). Expression of *SISTER OF ALTERED PHLOEM DEVELOPMENT* (*SAPL*), another CC marker gene^8^, also showed a strong correlation with *SUC2* expression (Fig. 2c; *r* = 0.91, *P* < 0.005). A microarray analysis using the same samples was performed to obtain a comprehensive gene expression profile (Supplementary Fig. 3a). As expected, genes previously characterized as CC-related, including *SULFATE TRANSPORTER 2;1* (*SULTR2;1*)^9^, *SODIUM POTASSIUM ROOT DEFECTIVE 1* (*NaKR1*)^10^, *C-TERMINALLY ENCODED PEPTIDE RECEPTOR* (*CEPR1*)*/XYLEM INTERMIXED WITH PHLOEM 1* (*XIP1*)^11,12^, and *MYB-RELATED PROTEIN 1* (*MYR1*)^13^, showed similar expression patterns to *SUC2* and *SAPL* (Fig. 2d). Consistently, quantitative RT-PCR assay for these genes validated the microarray result (Supplementary Fig. 4). By utilizing the variation in expression observed between samples (S, M, and V), we identified 186 VISUAL-CC-inducible genes that satisfied the following patterns of expression levels: S > M > V and S/V > 4 (Fig. 2e and Supplementary Fig. 2). According to the previous dataset of root cell-type-specific transcriptome^14^, these genes were mainly expressed in root CCs or phloem pole pericycles (PPPs, Fig. 2f). PPPs are also known to participate in phloem unloading from SEs in roots via intervening plasmodesmata^15^ (Fig. 2f). Here we grouped 67 genes as VISUAL-CC-related (VC) genes based on CC-preferential expression (Supplementary Table 1). Transporter genes were overrepresented among these VC genes, reflecting the functional aspect of phloem transport (Supplementary Fig. 3b, c).

### SE and CC differentiation shows a negative correlation

**Fig. 3:**
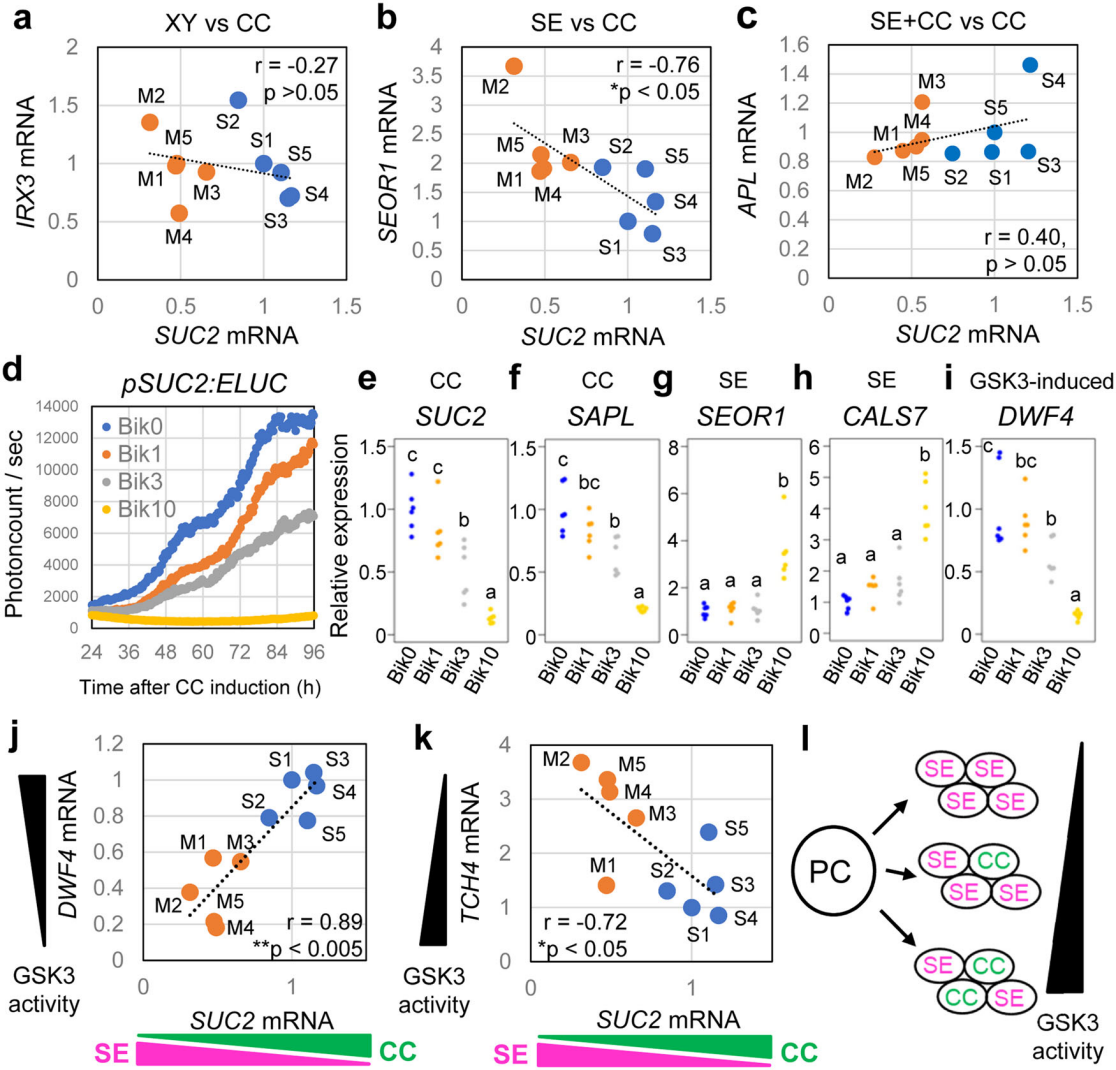
GSK3s activity balances the SE/CC ratio in VISUAL-CC. **a** Expression of *SUC2* (CC) and *IRX3* (XY) showing no correlation between levels in S and M samples. The Pearson correlation coefficient and *P* value are marked on the chart. **b** Negative correlation between expression of *SUC2* (CC) and *SEOR1* (SE). **c** Positive correlation between expression of *SUC2* (CC) and *APL* (SE + CC). **d** Time course of *pSUC2:ELUC* signal intensities during VISUAL-CC with various bikinin concentrations. Averaged LUC values are shown (*n* = 6). **e**–**i** Expression of *SUC2* (CC, **e**), *SAPL* (CC, **f**), *SEOR1* (SE, **g**), *CALS7* (SE, **h**), and *DWF4* (GSK3-induced, **i**) in VISUAL-CC samples cultured with various bikinin concentrations for 4 days. Dots indicate relative expression levels for each sample. Statistical differences between samples are indicated by different letters (ANOVA, Tukey–Kramer method, *n* = 6). **j** Positive correlation between expression of *SUC2* (CC) and *DWF4* (GSK3-induced). **k** Negative correlation between expression of *SUC2* (CC) and *TCH4* (GSK3-suppressed). **l** Schematic model showing dose-dependent regulation of the SE/CC ratio by GSK3 activity.

We previously identified 137 VISUAL-XY-related (VX) genes and 218 VISUAL-SE-related (VS) genes using VISUAL microarray data^2^. Then, expression of VX and VS genes was examined in the VISUAL-CC transcriptome dataset. Although there was no regular pattern of VX gene expression, expression of VS genes was very low in the S samples in contrast to that of VC genes (Fig. 2g–i). Correlation analysis among these gene sets revealed that expression levels of VC genes negatively correlate with those of VS genes (Supplementary Fig. 5b; *r* = −0.91, *P* < 0.05) but not with those of VX genes (Supplementary Fig. 5a; *r* = −0.28, *P* > 0.05). To further assess this tendency, we calculated the quantitative expression levels of vascular marker genes in individual samples. Although there was a strong correlation between expression of *SAPL* (CC) and *SUC2* (CC) (Fig. 2c, *r* = 0.91, *P* < 0.005), no correlation was found between *IRREGULAR XYLEM 3* (*IRX3*)^16^ (XY) and *SUC2* (CC) expression (Fig. 3a, *r* = −0.27, *P* > 0.05). By contrast, expression of *SEOR1* (SE) showed a significant negative correlation with that of *SUC2* (CC) (Fig. 3b, *r* = −0.76, *P* < 0.05). All these results suggest that CC and SE differentiation tend to show negative correlation. Interestingly, expression levels of *ALTERED PHLOEM DEVELOPMENT* (*APL*) (SE + CC), which is expressed in both SEs and CCs^17^, was almost constant among all the samples, indicating that the total amount of differentiating phloem cells is almost unchanged (Fig. 3c and Supplementary Fig. 6a). Taken together, these results suggest that VISUAL-CC induces different ratios of CC-like cells and SEs without changing the total number of phloem cells, while VISUAL only produced SEs. This implies that a key determinant of SE or CC cell fate is present in VISUAL-CC cultures.

### SE/CC cell fates mostly depend on GSK3 activity

To identify the determinants of SE/CC cell fate, we investigated the impact of auxin and bikinin, as their concentrations differed between VISUAL and VISUAL-CC media (Fig. 1c). Exogenous auxin reduced *pSUC2:ELUC* expression in a dose-dependent manner (Supplementary Fig. 7a). Although *SUC2* expression levels showed a similar decreased trend (Supplementary Fig. 7b), *SEOR1* expression was not affected by auxin level (Supplementary Fig. 7c). Similarly, bikinin showed a repressive effect on *pSUC2:ELUC* expression levels (Fig. 3d). Unlike auxin, treatment with bikinin decreased expression of *SUC2* in a dose-dependent manner while simultaneously increasing *SEOR1* expression (Fig. 3e–h). Furthermore, two other CC and SE markers, *SAPL* and *CALLOSE SYNTHASE 7* (*CALS7*)^18^ showed a similar response against bikinin treatment (Fig. 3e–h). Bikinin is a specific inhibitor of plant GSK3s^19^, and thus these results suggest that GSK3 activity plays a role in determining the SE/CC ratio. GSK3 activity correlates with the expression of BR biosynthetic genes, as a negative feedback regulation. Indeed, *DWARF4* (*DWF4*)^20^, one of BR biosynthetic genes, was downregulated following bikinin treatment in a dose-dependent manner (Fig. 3i). In the VISUAL-CC transcriptome, expression of BR biosynthetic genes tended to be higher in the S samples and lower in the M samples (Supplementary Fig. 8), also suggesting the relationship between the GSK3 activity and the SE/CC ratio. Previous studies have reported 113 bikinin-suppressed genes^19^; then we investigated the correlation between these genes and VC genes in VISUAL-CC transcriptome data. The bikinin-suppressed genes showed higher expression in the S samples (Fig. 2j) and their expression exhibited positive correlation with expression of VC genes (Supplementary Fig. 5c; *r* = 0.94, *P* < 0.01). To investigate the relationship further, we quantitatively compared the expression patterns of *SUC2* and GSK3-affected genes in the S and M samples. Expression of *DWF4*, a typical GSK3-induced gene (Fig. 3i), showed a strong positive correlation with *SUC2* expression (Fig. 3j, *r* = 0.89, *P* < 0.005). Similarly, other GSK3-induced genes such as *CONSTITUTIVE PHOTOMORPHOGENIC DWARF* (*CPD*) and *BRASSINOSTEROID-6-OXIDASE 2* (*BR6ox2*) showed significantly higher expression in the S samples than in the M samples^21,22^ (Supplementary Fig. 6b). By contrast, expression of *TOUCH 4* (*TCH4*), a typical GSK3-suppressed gene^23^, showed a significant negative correlation with *SUC2* expression (Fig. 3k, *r* = −0.72, *P* < 0.05). These results suggest that the ratio of induced SE and -CC is largely dependent on GSK3 activity in VISUAL-CC (Fig. 3l).

### Manipulation of GSK3 activity alters in vivo SE/CC ratio

**Fig. 4:**
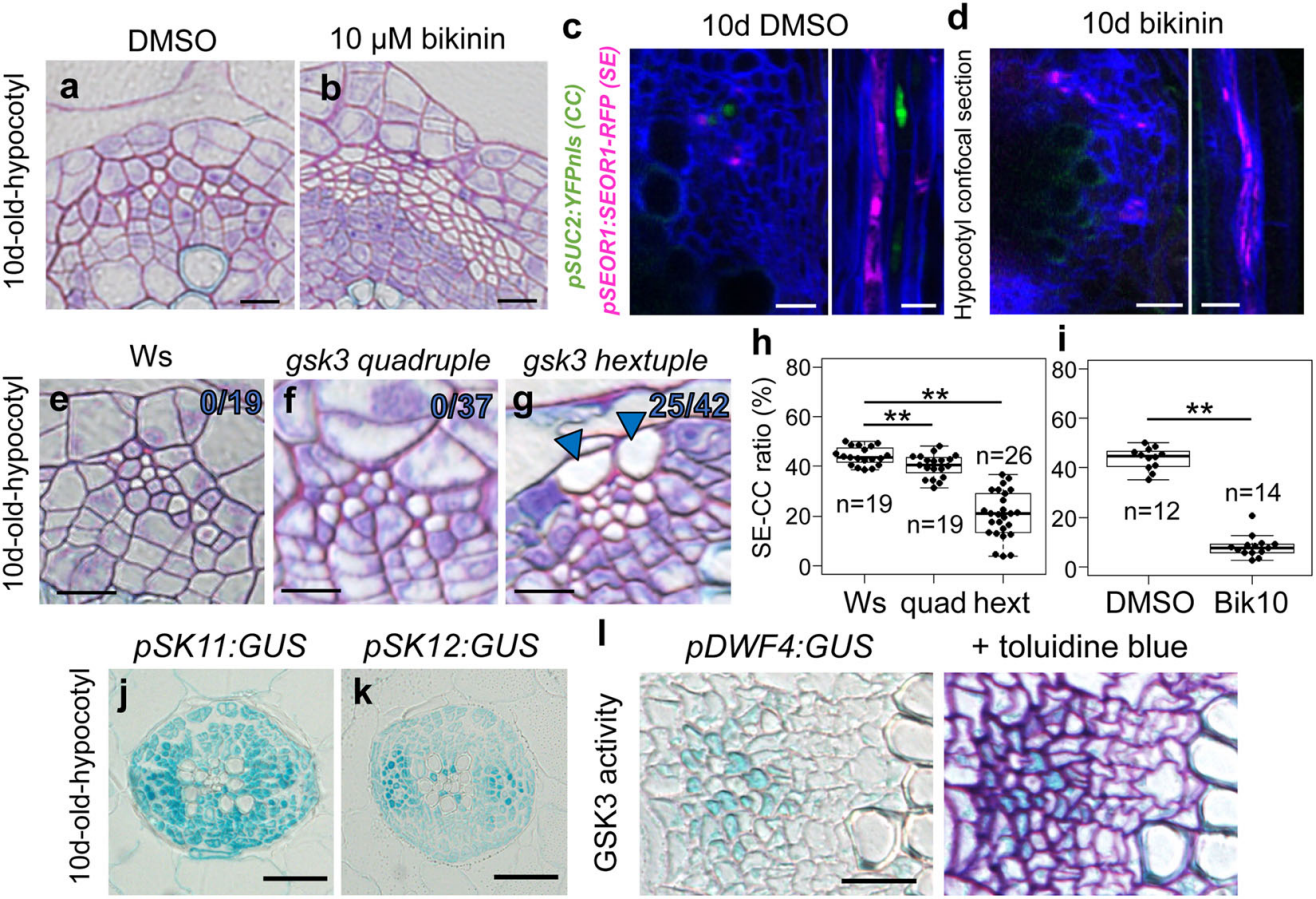
Reduction of GSK3 activity decreases the CC ratio in planta. **a**, **b** Toluidine blue-stained transverse sections through mock- (DMSO, **a**) and bikinin-treated (**b**) hypocotyls. SE, white empty cell; CC, dense purple cell. **c**, **d** Expression of *pSEOR1:SEOR1-RFP* (magenta) and *pSUC2:YFPnls* (green) in 10-day-old hypocotyls treated with DMSO (**c**) or 10 µM bikinin (**d**) for 10 days (*n* = 8). Confocal images of transverse (left) and longitudinal sections (right). **e**–**g** Toluidine blue-stained transverse sections for 10-day-old hypocotyls of Ws (wild type, **e** and *gsk3* high-order mutant plants (*gsk3 quadruple*, **f** and *gsk3 hextuple*, **g**). Arrowheads: ectopic SEs at the PPP position; the ratio of individuals showing ectopic SEs is marked on the upper right of the image. **h**, **i** Box-and-whisker plots of SE/CC ratios (%) in Ws and *gsk3* high-order mutants (**h**), and WT plants treated with or without 10 µM bikinin (**i**) calculated from toluidine blue-stained sections. Median values were indicated by central lines. The first (Q1) and third (Q3) quartile were shown as a box. Lines show the range of Q1 + 1.5× interquartile and Q3–1.5× interquartile. Dots indicated distributions of each sample. Numbers of individuals are marked (*n* = 12–26). Asterisks: significant differences determined using Dunnett’s or Student’s *t* test (**P < 0.005). **j**, **k** GUS staining of 10-day-old hypocotyls of *pSK11:GUS* (**j**) and *pSK12:GUS* (**k**) plants. **l** GUS staining of 11-day-old hypocotyls of *pDWF4:GUS* plants. GUS staining patterns on the cross section (left) and subsequent toluidine blue-stained section (right) were shown. GUS expression pattern was overlapped with toluidine blue staining. Scale bars: 10 µm (**a**–**g**); 20 µm (**j**, **k**); 50 µm (**l**).

Then, we analyzed the role of GSK3s in in vivo secondary phloem development in *Arabidopsis* hypocotyls. In hypocotyls, SEs are characterized by vacant cytoplasm, whereas CCs are deeply stained with toluidine blue and they usually appear as pairs in a transverse section (Fig. 4a). Inhibition of GSK3 activity by bikinin treatment induced clusters of SEs and far fewer CCs (Fig. 4b). Bikinin treatment consistently reduced expression of *pSUC2:YFPnls* and resulted in clusters of *pSEOR1:SEOR1-RFP* signals in the dual phloem marker line (Fig. 4c, d), indicating that bikinin promotes SE formation and decreased CC number in vivo. Next, we confirmed the function of GSK3 proteins genetically using knockout mutants of members of the SKII subfamily (*BIN2*, *BIL1*, and *BIL2*) and RNAi knockdown for SKI subfamily members (*AtSK11*, *AtSK12*, and *AtSK13*)^24^, because they are the main targets of bikinin^17^. The phloem tissue of the *bin2 bil1 bil2 AtSK13RNAi* quadruple mutant exhibited a slight but significant decrease in CC occupancy (40%) when compared with wild-type plants (44%) (Fig. 4e, f, h). The *gsk* hextuple mutant (*quadruple* + *AtSK11*, *AtSK12RNAi*) showed a reduction in CC occupancy (20%), resulting in more SEs and few CCs (Fig. 4g, h). Moreover, in the hextuple mutant, some of the PPP cells unexpectedly differentiated into ectopic SE-like cells (Fig. 4g). Previous studies have revealed that the vascular cells express SKII subgroup genes *BIN2* and *BIN2-LIKE2 (BIL2)*^24^. In addition, expression of SKI subgroup genes *pSK11:GUS* and *pSK12:GUS*^25^ was found in the vasculature, including the phloem tissue (Fig. 4j, k). Similarly to the GUS expression analysis, SKI/II gene expression was kept high in VISUAL time-course and in VISUAL-CC transcriptome data (Supplementary Fig. 9), indicating that six GSK3 members are present during phloem development. Next, we investigated local GSK3 activity in the vasculature using *pDWF4:GUS*, which is an indicator of high GSK3 activity. Supporting with our idea, *pDWF4:GUS* expression was detected in the phloem CCs but not in SEs (Fig. 4l). Taken together, our results indicate that GSK3 activity is required for maintaining high CC occupancy in planta.

**Fig. 5:**
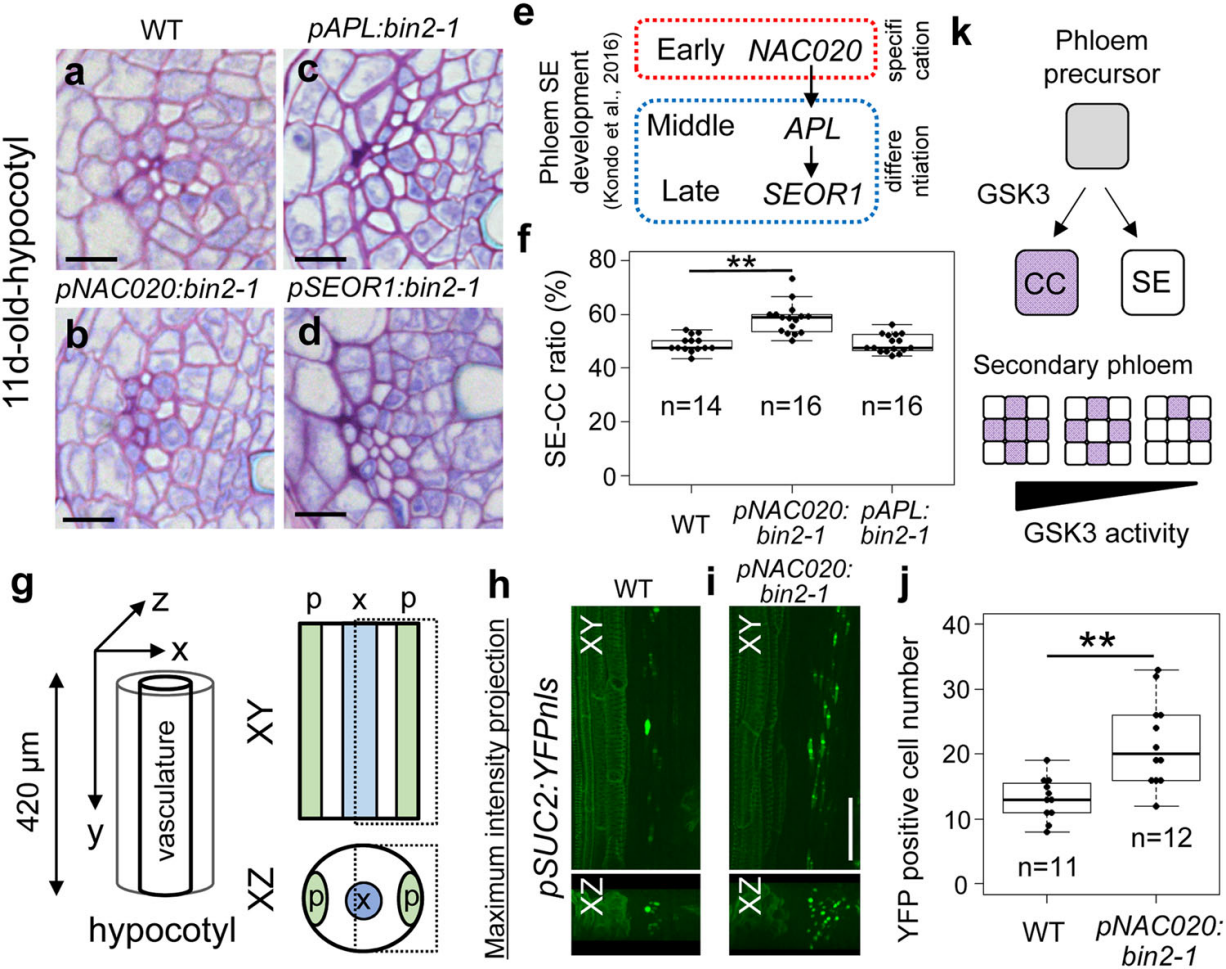
Early phloem-specific activation of GSK3 activity increases the CC cell number in planta. **a**–**d** Toluidine blue-stained transverse sections for hypocotyls of 11-day-old Col (wild type, **a**), *pNAC020:bin2-1* (**b**), *pAPL:bin2-1* (**c**), and *pSEOR1:bin2-1* (**d**) plants. **e** Genetic cascade in phloem development revealed by VISUAL. **f** Box-and-whisker plots of SE/CC ratios (%) in wild type (WT), *pNAC020:bin2-1*, *pAPL:bin2-1*, and *pSEOR1:bin2-1* calculated from toluidine blue-stained sections (*n* = 14–16). **g** Schematic illustration for 3D confocal imaging of *pSUC2:YFPnls* marker expression in hypocotyls. **h**, **i** Maximum intensity projection of *pSUC2:YFPnls* marker expression in the WT (**h**) and *pNAC020:bin2-1* (**i**) was shown from XY (upper) and XZ (lower) angles. **j** Quantification of *pSUC2:YFPnls*-positive cells from 3D-reconstruction images in the WT and *pNAC020:bin2-1* (*n* = 11–12). Asterisks: significant differences determined using Student’s *t* test (**P < 0.005). **k** Schematic showing that GSK3 activity determines the SE/CC ratio. For box-and-whisker plots, median values were indicated by central lines. The first (Q1) and third (Q3) quartile were shown as a box. Lines show the range of Q1 + 1.5× interquartile and Q3 − 1.5× interquartile. Dots indicated distributions of each sample. Scale bars: 10 µm (**a**–**d**); 50 µm (**h**, **i**).

GSK3s function as signaling hubs to control xylem differentiation in the cambium^24^. Here to focus on phloem development, *bin2-1*, a stable form of GSK3^26^, was driven under promoters specific to each stage of phloem development. As we had previously demonstrated that the sequential genetic cascade in phloem SE differentiation is *NAC020* (early), *APL* (middle), *SEOR1* (late)^2^, we induced expression of *bin2-1* under these different phloem promoters and investigated their phloem phenotype (Fig. 5a–f). Expression of *bin2-1* driven by the *APL* and *SEOR1* promoters did not affect phloem phenotypes, but *pNAC020:bin2-1* slightly increased the ratio of CCs in the phloem (Fig. 5a–f). To objectively confirm the results with the CC marker, the number of *pSUC2:YFPnls* signal in WT and *pNAC020:bin2-1* was quantified using a confocal microscope (Fig. 5g). YFP-positive cell number estimated from 3D-reconstruction images was significantly higher in the *pNAC020:bin2-1* than in the WT (Fig. 5h–j), indicating that activation of GSK3 in the early phloem leads to increment of CC cell number. Taken together, our results suggest that GSK3s appear to function as cell-fate switches for determining differentiation into phloem CCs or SEs, and that GSK3 activity will be important for ensuring the proper ratio between CCs and SEs (Fig. 5k).

### BR-BES1 signaling is not involved in SE/CC-fate regulation

Finally, we examined the involvement of BR in CC differentiation, because GSK3s function as signal mediators in BR signaling^26,27^. However, application of brassinolide (BL), an active BR, did not alter the SE/CC ratio in hypocotyls (Supplementary Fig. 10). Moreover, *bes1 bzr1* loss-of-function and *bes1-D bzr1-D* gain-of-function mutants for *BRI1-EMS-SUPPRESSOR 1* (*BES1*) and *BRASSINAXOLE RESISTANT 1* (*BZR1*), which are well-known transcription factors phosphorylated by GSK3s in BR signaling^28^, exhibited a normal phloem development in terms of the SE/CC ratio (Supplementary Fig. 11). These results suggest the possibility that other signaling pathway(s) than BR participates in controlling the SE/CC ratio.

## Discussion

Recent studies have revealed that CCs are not only of importance for phloem transport, but also act as a signal center integrating environmental information into the developmental program^29^. We have established VISUAL-CC as a powerful tool for analyzing the functional and developmental processes of CCs. During secondary vascular development, it has been widely believed that SEs and CCs are derived from the same phloem precursors via asymmetric cell division^30^. In VISUAL-CC, SEs and CCs were formed from vascular stem cells as neighboring complexes after several rounds of cell division, suggesting that VISUAL-CC may reflect the process of secondary phloem development. By taking advantages of VISUAL-CC, we identified GSK3s as key molecular switches to specify cell fate toward SE or CC. Indeed, *gsk3* gain-of-function and loss-of-function mutants altered the ratio of SE/CC differentiation in the hypocotyl vasculature, and our expression analysis together with previous results consistently revealed that SKI/II subgroup members of GSK3s including BIN2 are expressed in the vascular tissues of hypocotyls^24^. As SEs lose their nuclei during the differentiation process, support from adjacent CCs is essential for their function. Thus, maintenance of the SE/CC ratio by GSK3s will be an important mechanism ensuring survival under various environmental conditions. Although GSK3s act as a central regulator of SE/CC development, genetic experiments suggested the involvement of other signaling than BR mediated by GSK3s in this regulation. GSK3s have been implicated in regulation of phloem development through the interaction with OCTOPUS (OPS), which genetically functions together with BREVIS RADIX (BRX)^31^. Further studies on such interacting proteins and reverse genetic approaches combined with VISUAL-CC transcriptome data will be helpful for elucidating a new signaling cascade controlling the SE/CC ratio.

GSK3s also function in animals as molecular switches determining differentiation into alternate cell types^32^, suggesting their common and important role as cell-fate switches. On the other hand, GSK3 activity regulates asymmetric cell division in the stomata lineage by interacting with polarly localized proteins, which are required for specifying stomatal cell fate^33^. Further investigation with the context of asymmetric cell division will uncover the extent to which GSK3s serve as a common mechanism determining cell fate.

## Methods

### Plant materials

*Arabidopsis* plants used in this study is Col-0 accession, except for *gsk3* high-order mutants (Ws background). To construct the CC-reporter lines, approximately 2.0 kb of the *SUC2* promoter region was cloned and then fused with ELUC (Toyobo) or YFP containing a nuclear localization signal. A genomic fragment of *SEOR1*, approximately 4.8-kb long and containing 1.6 kb of the promoter, was fused with RFP to make *pSEOR1:SEOR1-RFP*; this was subsequently transformed into *pSUC2:YFPnls* to generate the double phloem marker line. To construct *pDWF4:GUS*, approximately 1.9 kb of the *DWF4* promoter region was cloned and introduced into pMDC163 vector to create a *GUS*-fusion gene.

Phloem-specific GSK3 activation lines were constructed by cloning *bin2-1* (*BIN2E263K*) and fusing it with the *NAC020* (2.4 kb), *APL* (2.9 kb), and *SEOR1* (1.6 kb) promoters using the LR reaction (Thermo Fisher Scientific). The *gsk quadruple* and *hextuple* mutants (Ws accession) used in this study were as reported previously^23^. The *smxl4 smxl5* mutants were as reported previously^6,7^. The *bes1 bzr1* loss-of-function mutants and *bes1-D bzr1-D* gain-of-function mutants were as reported previously^34^. *pSK11:GUS* and *pSK12:GUS* lines were as reported previously^25^.

### Luciferase measurement

In this study, ELUC with PEST domain (Toyobo) was used as a short half-life luminescent protein. *pSUC2:ELUC* seedlings were co-cultured with 200 µM d-luciferin (Wako) in white 24-well plates (PerkinElmer). The time course of luciferase (LUC) activity was measured automatically using a TriStar2 LB942 (Berthold) within a growth chamber (Nihonika).

### Microscopic observation

For deep imaging with confocal microscopes, isolated tissue samples were fixed for 3 h under vacuum in a fixative solution (4% paraformaldehyde and 0.01% Triton X-100 in 1× phosphate-buffered saline (PBS)). Fixed samples were washed twice with 1× PBS and transferred to ClearSee solution (25% urea, 15% xylitol, and 10% sodium deoxycholate). ClearSee solution was replaced with fresh solution every 2 days for 3–4 weeks. Calcofluor staining was performed 1 week before microscopic observations by adding 0.1% (w/v) calcofluor white to the ClearSee solution. The samples were stained overnight and then washed with ClearSee solution without calcofluor. Once the samples were stained, washing was continued as described above. Cleared samples were observed using LSM880 (Zeiss) or FV1200 (Olympus) confocal microscopes with Z stack. For the quantification of YFP-positive cells, we counted the number of cells in a phloem pole of approximately 420-µm length of hypocotyls based on reconstructed 3D images.

### Electron microscopy

Sample preparation for electron microscopy observation was modified slightly from a previous study^35^. Briefly, leaf disks induced by VISUAL-CC were fixed and embedded in resin. Thin sections (100 nm) were mounted on glass slides. Sections were stained with 0.4% uranyl acetate solution (UA) and a lead citrate solution (Pb), and then coated with osmium tetroxide. Observations of slides were made using a FE-SEM (Hitachi SU 8220). Thinner (80 nm) sections were mounted on formvar-coated 1-slot copper grids, stained with 4% UA and Pb, and then observed using an 80-kV transmission electron microscope (JEOL JEM-1400 Flash).

### qRT-PCR and microarray experiments

Total RNA was extracted from four cotyledons using RNeasy plant mini kit (Qiagen) after LUC measurement. After reverse transcription reaction, qRT-PCR was performed using LightCycler 480II (Roche) by a universal probe method. The expression value was normalized with an internal control *UBQ14*. Microarray experiments were conducted with the Arabidopsis Gene 1.0 ST Array (Affymetrix) and analyzed with Subio platform and R gplots package. Primers used in this study are listed in Supplementary Table 2.

### Cross-section

Hypocotyls of 10- or 11-day-old seedlings were fixed with FAA (formalin:acetic acid:alcohol, 1:1:18) for 1 day. Fixed hypocotyls were subjected to an ethanol series (50%, 70%, 80%, 90%, and 99.5%) each for 30 min and then transferred into Technovit 7100 solution without Hardener II for 1 day. After the preincubation, samples were embedded in a mixture of Technovit 7100 + Hardener II (12.5:1) and incubated at 37 °C for more than 1 h to harden. Technovit samples were sliced into 2-µm sections using a LEICA RM2255 microtome and stained with 0.1% toluidine blue to enable CCs (deep purple) to be distinguished from SEs (white) under microscopy. We counted the CC/SE cell number per phloem pole on the sections and statistically analyzed the difference using more than 12 samples. Cross sections for GUS-stained samples were made as reported previously^24^.

### Statistics and reproducibility

Microarray analysis was performed using three biologically independent samples for S, M, and V. Correlation among the samples was analyzed by Pearson correlation coefficient. For the quantification of cross-section or confocal images, at least two biologically independent samples were used. Statistical differences were computed by Student’s *t* test or ANOVA (Tukey–Kramer method).

### VISUAL-CC

This protocol was a modified one from the previous VISUAL method^36^. VISUAL-CC consists of two distinct steps: vascular stem cell formation and subsequent phloem differentiation. As the initial step, 6- or 7-day-old seedlings were cultured with the conventional VISUAL medium for 2 days in order to induce sufficient amount of (pro)cambial cells. After that, samples are transferred into VISUAL-CC medium for SE–CC complex differentiation.

#### Materials for growth of plant samples before VISUAL induction

1. MS growth medium: It contains 2.2 g/L MS Basal Medium (Sigma), 10 g/L sucrose, and 0.5 g/L 2-morpholinoethanesulfonic acid monohydrate (MES) in Milli-Q water and the pH is adjusted to 5.7 with KOH. The solution is autoclaved at 120 °C for 20 min and can be stored at room temperature up to several weeks.
2. Sterilizing solution: Sodium hypochlorite solution is diluted in Milli-Q water in the ratio 1:9 (v/v) and 0.1% of Triton X-100 is added. This solution is prepared immediately before the sterilizing procedure.
3. Sterilized 6-well plate (Sumilon).
4. Autoclaved Milli-Q water.
5. Surgical tape.
6. Continuous light chamber (22 °C, 45–55 µmol/m^2^/s).
7. Rotary shaker (Taitec).

#### Materials for VISUAL and VISUAL-CC

1. VISUAL base medium: It contains 2.2 g/L MS Basal Medium and 50 g/L D(+)-Glucose in Milli-Q water and the pH is adjusted to 5.7 with KOH. The solution is autoclaved at 120 °C for 20 min and can be stored at room temperature for several weeks.
2. VISUAL-CC base medium: It contains 2.2 g/L MS Basal Medium and 10 g/L D(+)-Glucose in Milli-Q water and the pH is adjusted to 5.7 with KOH. The solution is autoclaved at 120 °C for 20 min and can be stored at room temperature for several weeks. Note that Glucose concentration is different from that of VISUAL base medium.
3. 2,4-D stock: About 2.5 g/L 2,4-D stock dissolved in autoclaved Milli-Q water and sterilized through 0.22-µm filter units. It is stored in small amounts in sampling tubes at −20 °C.
4. Kinetin stock: About 0.5 g/L Kinetin stock dissolved in 0.1 M KOH and sterilized through 0.22-µm filter units. It is stored in small amounts in sampling tubes at −20 °C.
5. Bikinin stock: About 10 mM Bikinin stock dissolved in DMSO and sterilized through 0.22-µm filter units. It is stored in small amounts in sampling tubes at −20 °C.
6. Sterilized 12-well plate (Sumilon).
7. Surgical forceps.
8. Continuous light chamber (22 °C, 60–70 µmol/m^2^/s).
9. Rotary shaker (Taitec).

#### Methods for growth of plant samples before VISUAL induction

1. Sterilizing solution is added to the *Arabidopsis* seeds in 1.5-mL sampling tubes and gently mixed using a rotator for 5 min. The tubes are transferred inside a clean bench and allowed to stand for further 5 min. The sterilizing solution is then removed using a pipette, and the seeds are washed with autoclaved Milli-Q water three times. The seeds are soaked in water at 4 °C for 2 days to keep the germination timing constant.
2. About 10 mL of the prepared MS growth medium is poured into each well of a 6-well plate. Seeds are sown at a density of 8–10 seeds/well containing the MS growth medium, and the plate is sealed with surgical tape. The well plate is incubated for 6–7 days under continuous light (22 °C, 45–55 µmol/m^2^/s) with shaking at 110 rpm on a rotary shaker.

#### Methods for VISUAL

1. 2,4-D stock, kinetin stock, and bikinin stock are defrosted at room temperature before use. The tubes are transferred inside a clean bench and added to the VISUAL base medium to obtain a final concentration of 1.25 mg/L 2,4-D, 0.25 mg/L kinetin, and 10 µM bikinin. About 2.5 mL of the above medium is then added into each well of a 12-well plate.
2. A pair of sharp surgical forceps are used to cut the bottom half of *Arabidopsis* 6–7-day-old plants across the center of the hypocotyl and the roots are removed. About 4 of the *Arabidopsis* explants are then transferred carefully to each well containing the induction medium using forceps, and the 12-well plate is sealed with surgical tape. The explants are cultured for 2 days under continuous light (22 °C, 60–70 µmol/m^2^/s) with shaking at 110 rpm on a rotary shaker.

#### Methods for VISUAL-CC

1. 2,4-D stock, kinetin stock, and bikinin stock are defrosted at room temperature before use. The tubes are transferred inside a clean bench and added to the VISUAL-CC base medium to obtain a final concentration of 0.25 mg/L 2,4-D, 0.25 mg/L kinetin, and 1 µM bikinin. About 2.5 mL of the above medium is then added into each well of a 12-well plate. Note that auxin and bikinin concentration is decreased when compared with the VISUAL.
2. VISUAL-induced samples were transferred into the new CC medium and then cultured for 4 days under dark conditions (22 °C) with shaking at 110 rpm on a rotary shaker. Note that light severely affects the CC differentiation ratio.

### Reporting summary

Further information on research design is available in the Nature Research Reporting Summary linked to this article.

## Supplementary information

Supplementary Information

Supplementary Data 1

Description of Additional Supplementary Files

Reporting Summary

Peer Review File

## Data Availability

Accession number of microarray data for VISUAL-CC is GSE141037. The Supplementary information includes the 67 VC genes list characterized in this study as Supplementary Table 1.
