# Supplementary material for: VISUAL-CC system uncovers the role of GSK3 as an orchestrator of vascular cell type ratio in plants
Source: Commun Biol. 2020 Apr 22;3:184. doi: 10.1038/s42003-020-0907-3 (PMC7176705; doi:10.1038/s42003-020-0907-3)
Supplement: Supplementary file 1 — Supplementary Information [file 42003_2020_907_MOESM1_ESM.pdf]

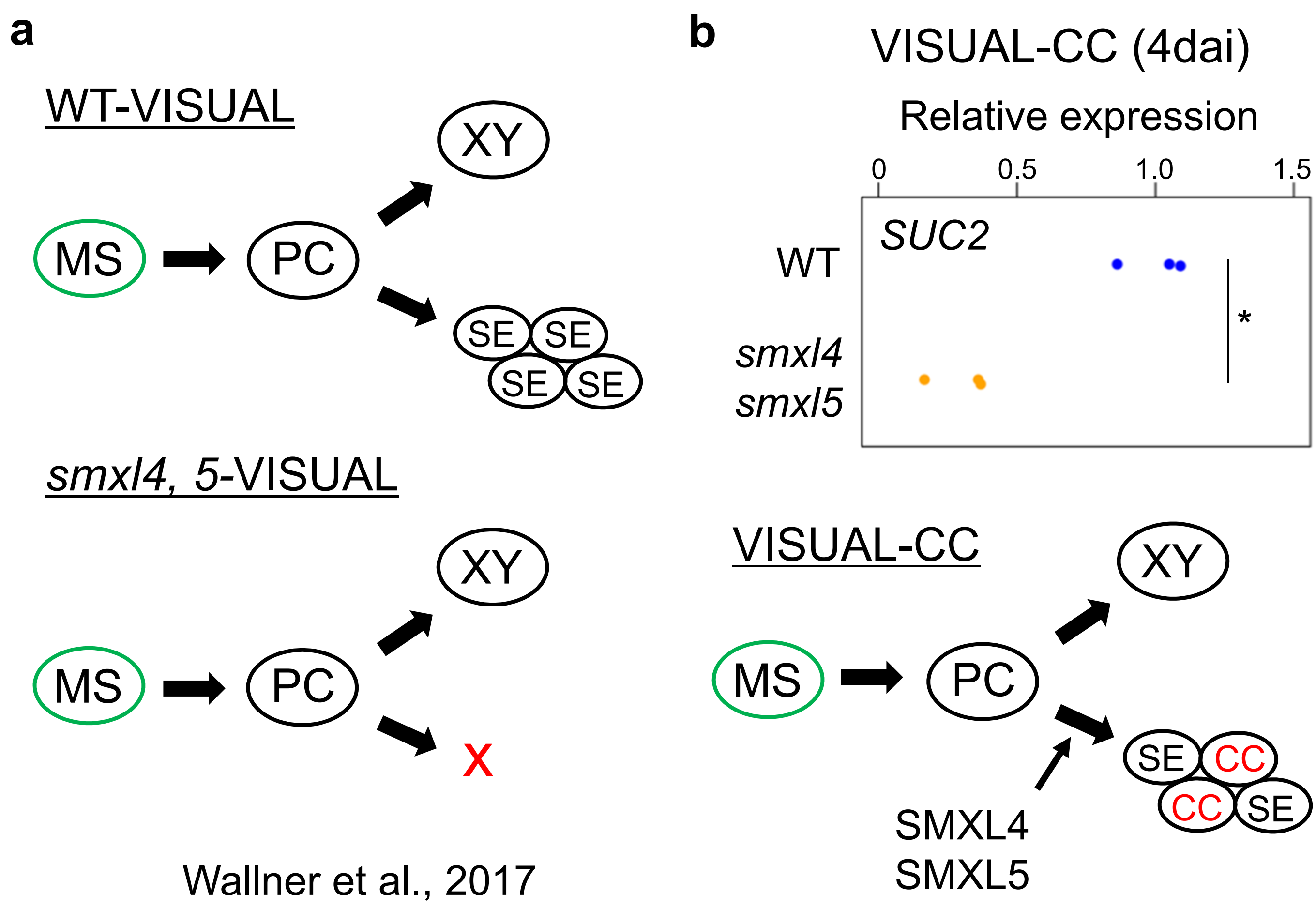

Supplementary Fig. 1| *smxl4 smxl5* mutants suppressed CC differentiation in VISUAL-CC

a, Schematic of the VISUAL differentiation process in the WT and *smxl4 smxl5*. The *smxl4 smxl5* double mutants were known to inhibit phloem differentiation in VISUAL. **b**, SUC2 expression at 4d after VISUAL-CC induction in the WT and *smxl4 smxl5*. Asterisks indicate significant differences using the Student's t-test (**P* < 0.05, n = 3).

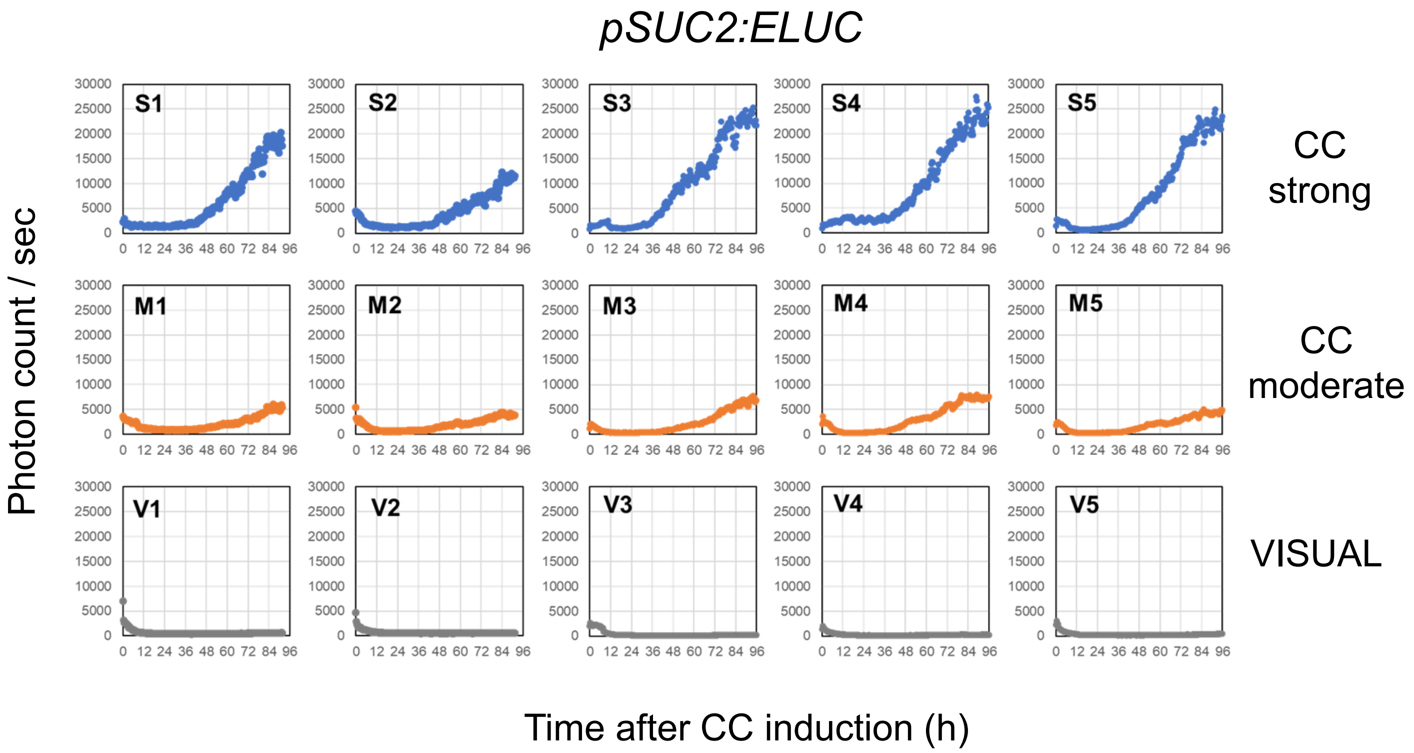

Supplementary Fig. 2| Raw data from time-course analysis of *pSUC2:ELUC* plants

An example of *pSUC2:ELUC* signals from individual samples is shown. Vertical axis indicates photon counts per second detected by the luminometer. Samples were classified based on LUC intensity.

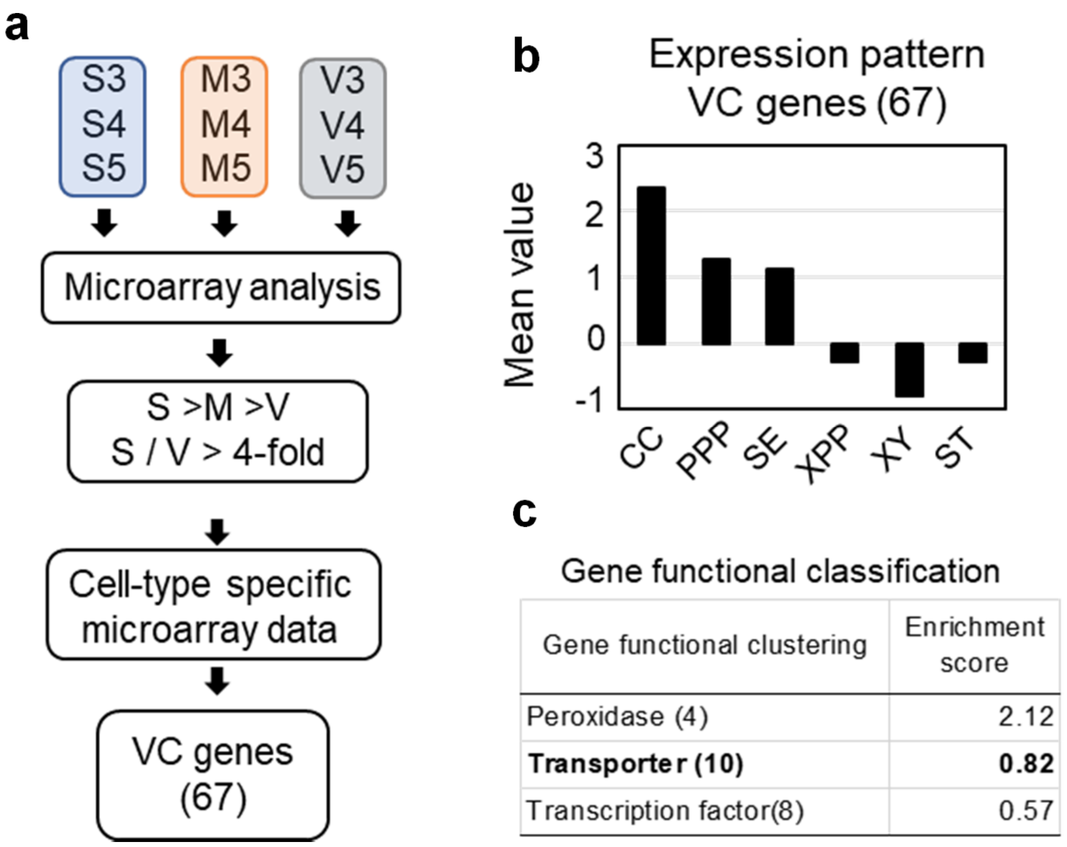

Gene functional clustering	Enrichment score
Peroxidase (4)	2.12
**Transporter (10)**	**0.82**
Transcription factor(8)	0.57

Supplementary Fig. 3| Characterization and molecular function of VC genes

a, Schematic of the selection process used to identify VISUAL-CC inducible genes. Expression levels of vascular-specific genes were determined using VISUAL-CC microarray data. **b**, Expression patterns of VC genes in the root stele obtained from a transcriptome dataset[14]. Mean values from Fig. 2A are shown. **c**, Functional classification of VC genes and VPP genes. Enrichment scores were calculated using David (https://david.ncifcrf.gov/). Transporter-related genes are over-represented in this category.

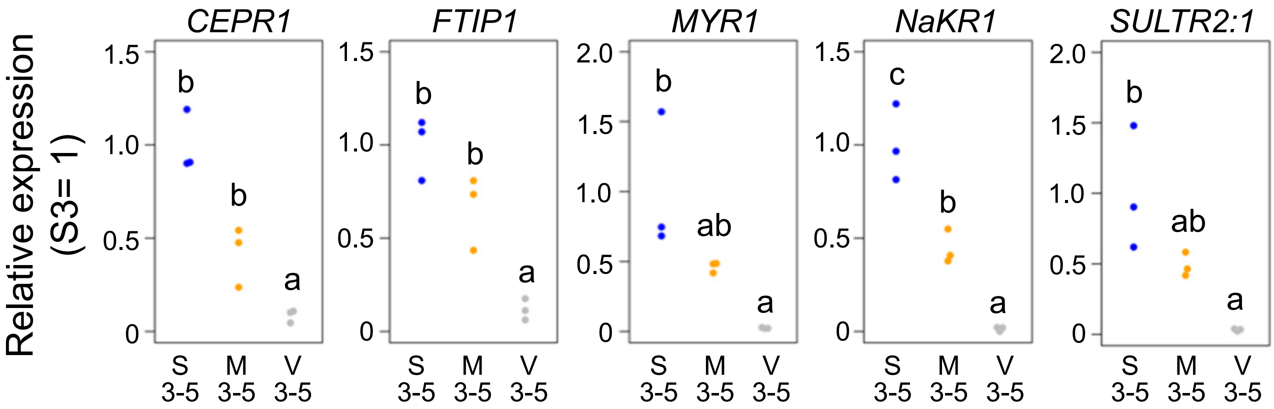

Supplementary Fig. 4 | Statistical differences in expression levels of CC-related genes among the S, M, and V samples

Expression levels of *CEPR, FTIP1, MYR1, NaKR1,* and *SULTR2:1* were confirmed using qRT-PCR and compared statistically among the S3-5, M3-5, and V3-5 samples. Relative expression levels were calculated when the expression in S3 was set to 1. Statistical differences between samples are indicated by different letters (ANOVA, Tukey-Kramer method; n = 3; error bars indicate SD).

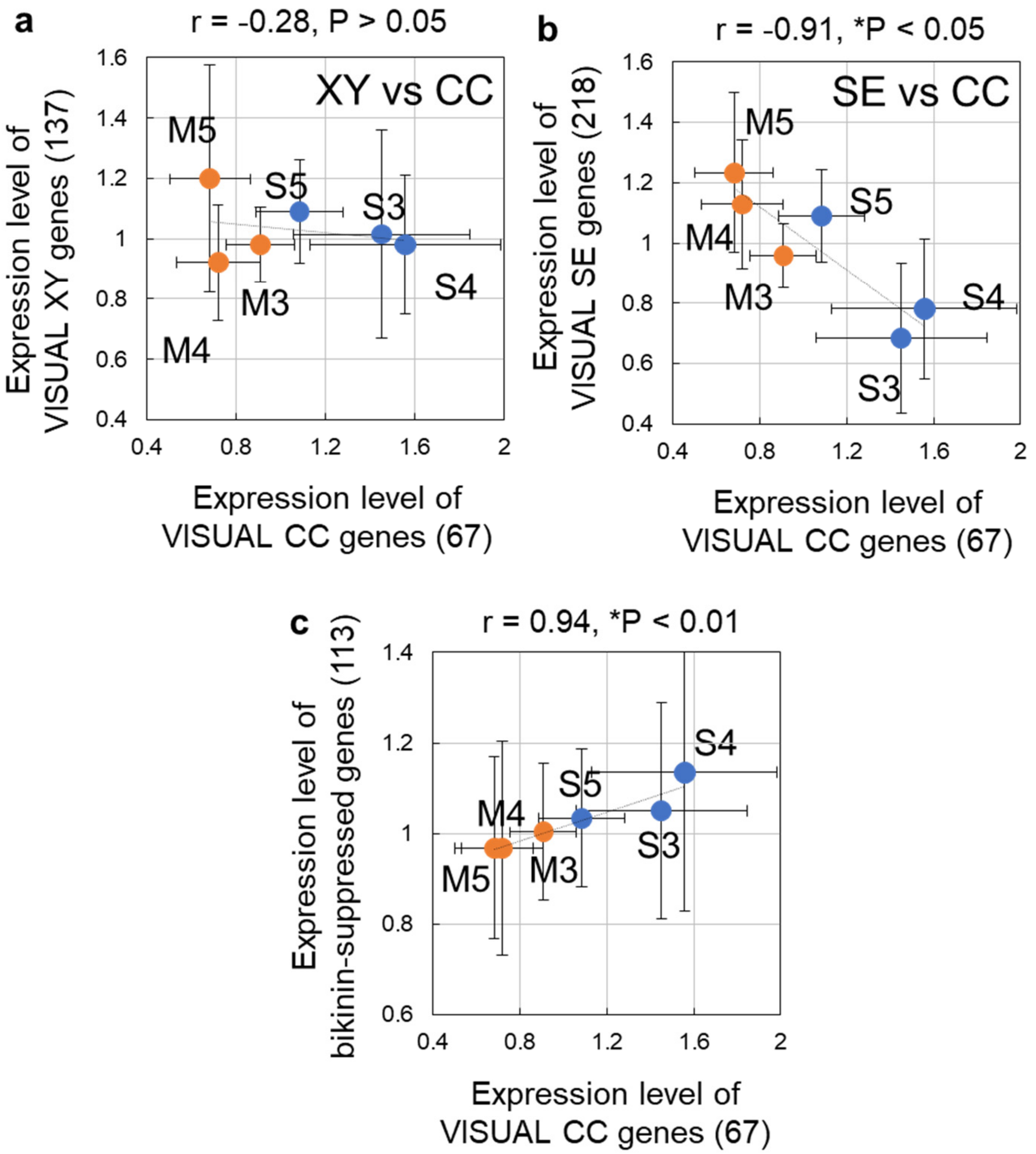

Supplementary Fig. 5 | Correlation analysis in microarray data between the S and M samples

a, VC genes (67) vs VX genes (137) **b**, VC genes (67) vs VS genes (218) **c**, VC genes (67) vs bikinin-suppressed genes (113). The Pearson correlation coefficient and *P*-value are marked above the chart. Error bars indicate SD.

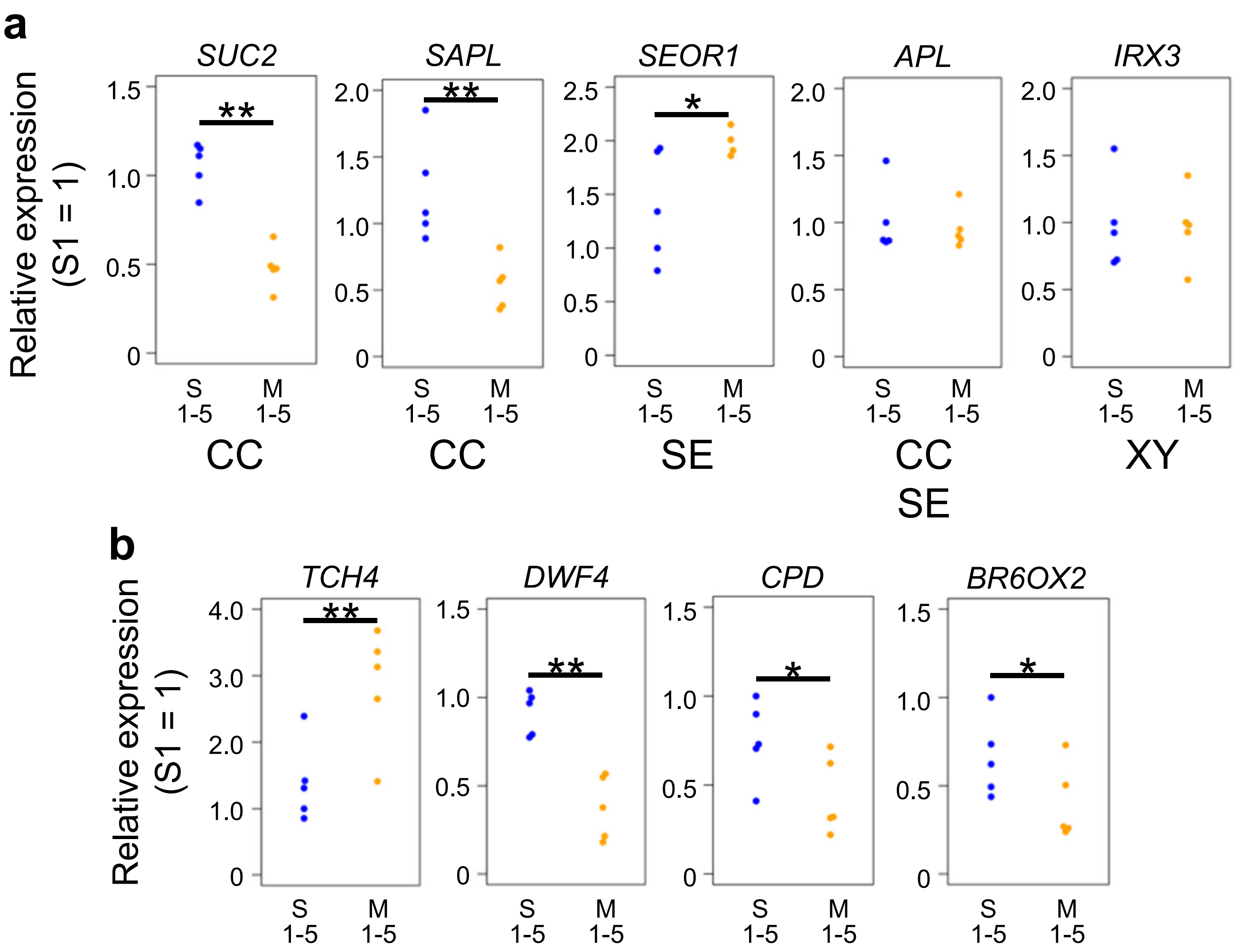

Supplementary Fig. 6| Statistical differences in expression levels between the S and M samples

a, Expression levels of *SUC2* (as CC), *SAPL* (also as CC), *SEOR1* (as SE), *APL* (as CC+SE), and *IRX3* (XY) were quantified using qRT-PCR and compared statistically between the S and M samples. Asterisks indicate significant differences using the Student's t-test (**P < 0.005; *P < 0.05). **b**, Expression levels of GSK3 activity-dependent genes were quantified using qRT-PCR and compared statistically in the S and M samples. Asterisks indicate significant differences determined using the Student's t-test (**P < 0.005; *P < 0.05).

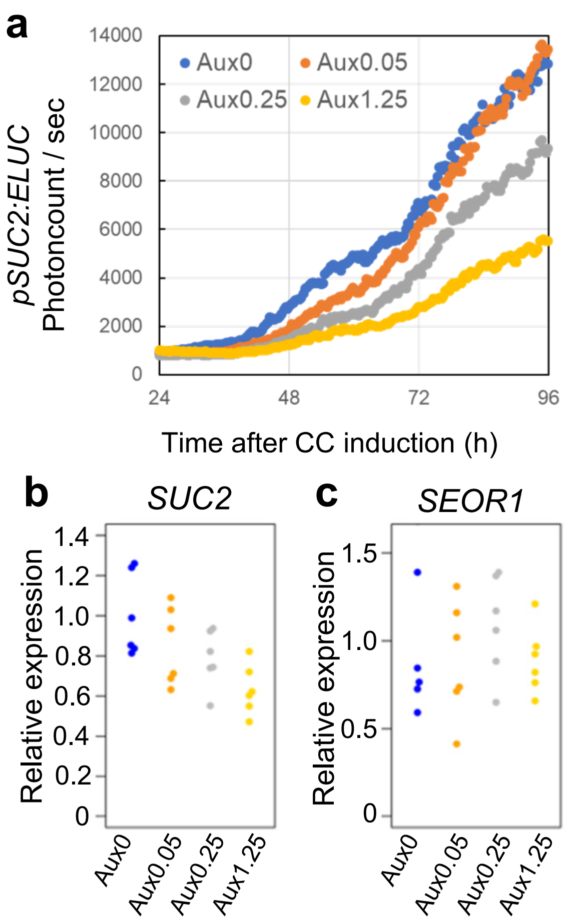

Supplementary Fig. 7| Auxin has only marginal effects on the formation of the SE-CC complex

a, Time-course of *pSUC2:ELUC* signal intensities in VISUAL-CC cultures containing different concentrations of auxin (mg/L). **b** and **c**, Expression levels of *SUC2* (b) and *SEOR1* (c) in VISUAL-CC samples from cultures containing different concentrations of auxin. There are no significant differences (ANOVA, Tukey-Kramer method; n = 6; error bars indicate SD).

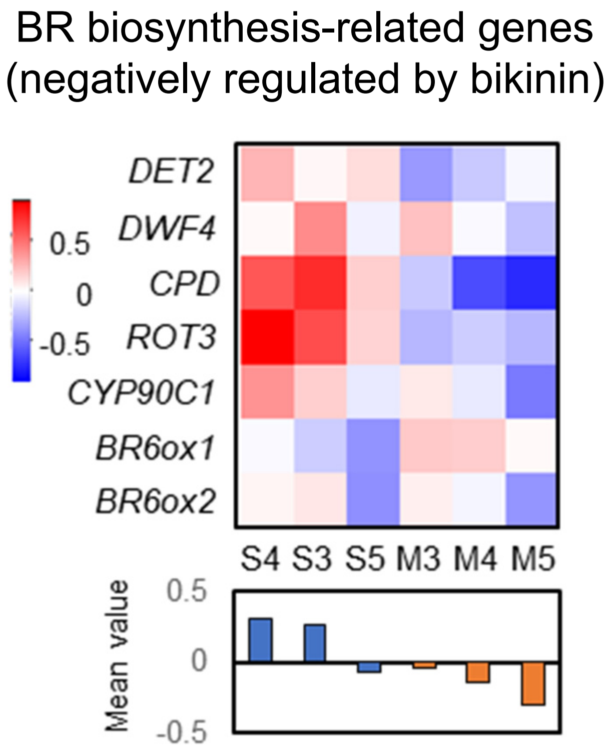

Supplementary Fig. 8| Heat map of expression levels of BR biosynthesis-related genes in S and M samples.
The upper panel shows a heat map of expression levels of 6 BR biosynthesis-related genes, which are downregulated by bikinin, in S and M samples. The lower panel indicates the mean value for each sample.

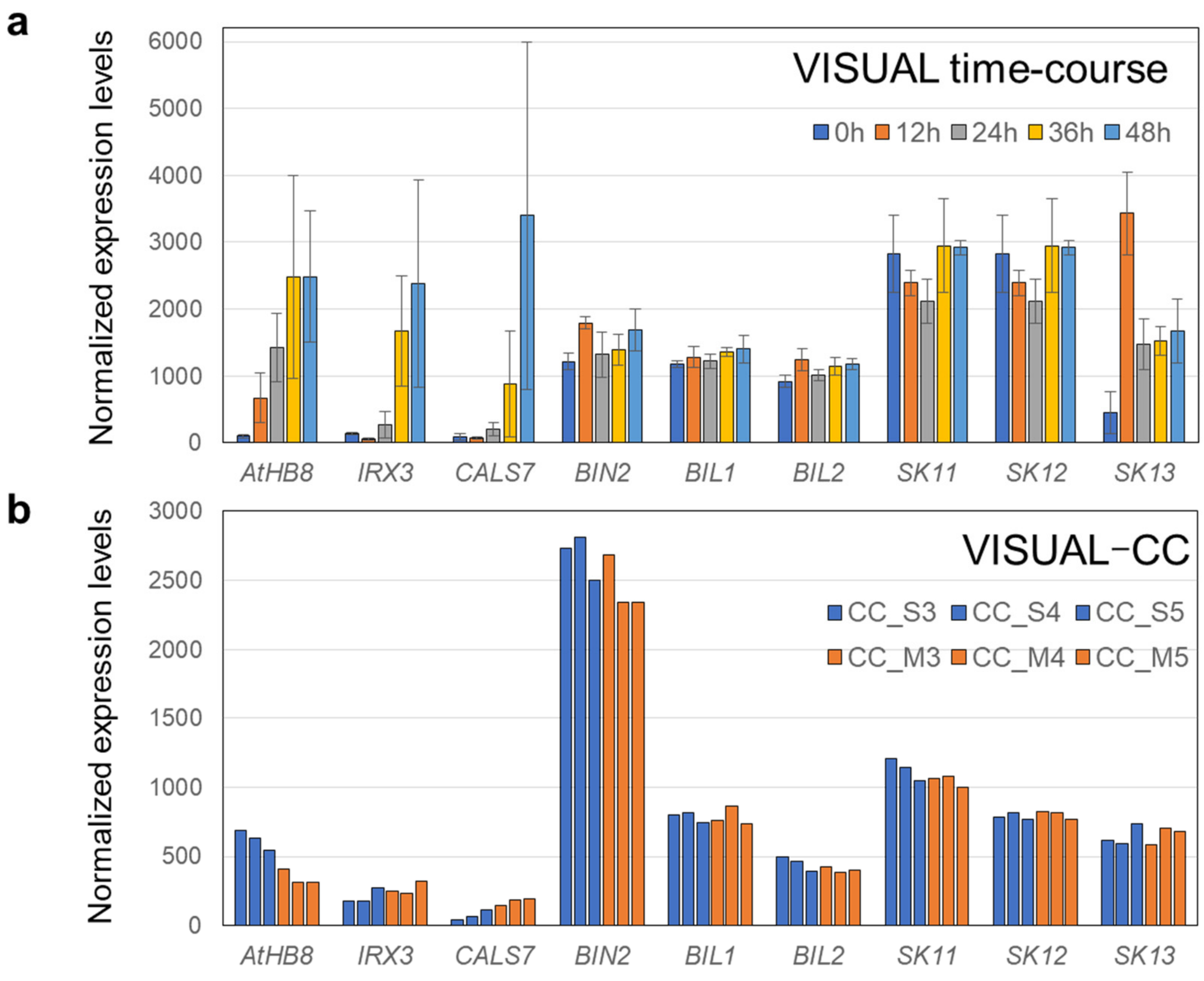

Supplementary Fig. 9| Expression pattern of GSK3s in VISUAL and VISUAL-CC transcriptome data

a, Normalized expression levels of procambium (*AtHB8*), xylem *(IRX3*), phloem SE (*CALS7*) and SKI/II GSK3 subgroup genes in VISUAL transcriptome data. Error bars indicate SD (n=3). **b,** Normalized expression levels of procambium (*AtHB8*), xylem *(IRX3*), phloem SE (*CALS7*) and SKI/II GSK3 subgroup genes in VISUAL-CC transcriptome data.

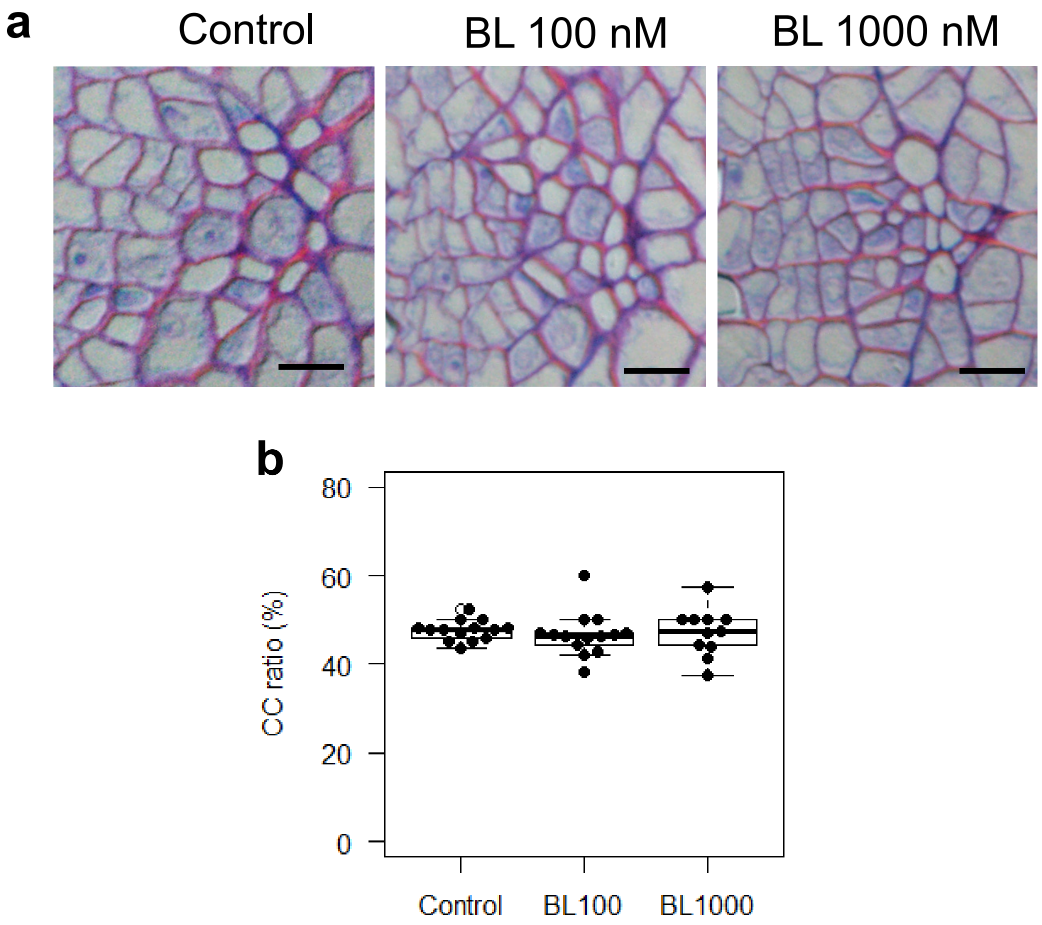

Supplementary Fig. 10| Effect of brassinolide treatment on phloem development

a, Toluidine blue-stained transverse sections of mock-treated (DMSO) and bikinin-treated hypocotyls. SE: white empty cell; CC: dense purple cell. **b**, SE/CC ratios (%) in the WT treated with none (control), 100 nM BL, and 1000 nM BL were calculated from toluidine blue-stained sections (n = 11–14). Numbers of individuals are marked. Scale bars: 10 μm.

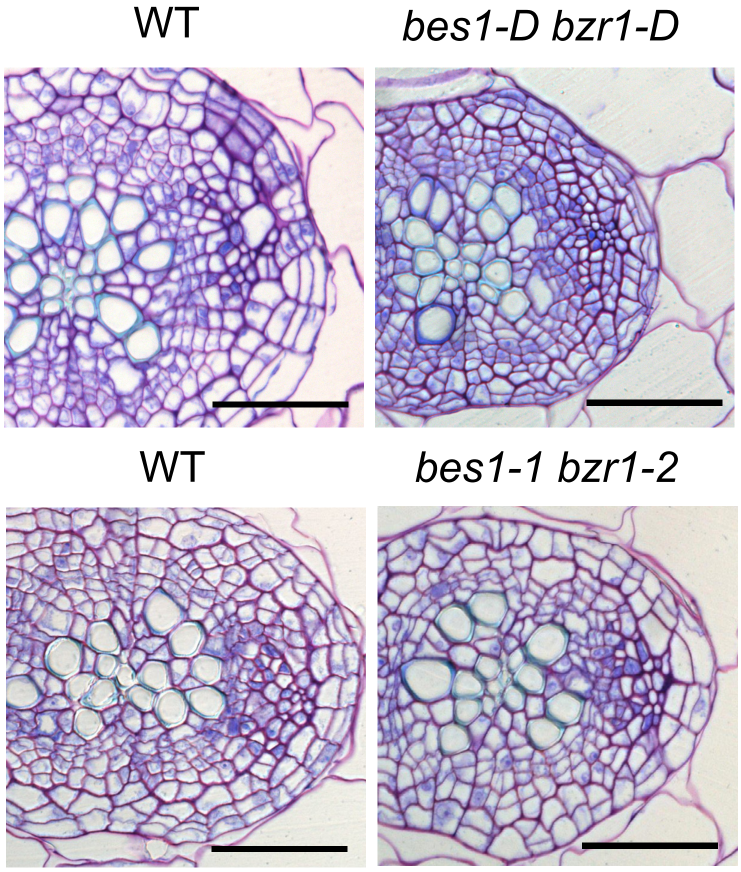

Supplementary Fig. 11| Transverse sections of *bes1 bzr1* mutants

Toluidine blue-stained transverse sections for 11-day-old hypocotyls of WT, *bes1-D bzr1-D* (gain-of-function), and *bes1-1 bzr1-2* (loss-of-function) mutant plants. Scale bars: 50 µm.

Supplementary Table 1. The list of VC genes (67).

AGI code	Description (based on TAIR)
At1g01470	Late embryogenesis abundant protein (LEA14)
At1g10380	Putative membrane lipoprotein
At1g12090	extensin-like protein (ELP)
At1g13380	Protein of unknown function (DUF1218) (DUF1218)
At1g13590	phytosulfokine 1 precursor (PSK1)
At1g22710	sucrose-proton symporter 2 (SUC2)
At1g49310	transmembrane protein
At1g49500	transcription initiation factor TFIID subunit 1b-like protein
At1g59740	NRT1/ PTR FAMILY 4.3
At1g59960	NAD(P)-linked oxidoreductase superfamily protein
At1g68740	PHO1;H1
At1g76130	alpha-amylase-like 2 (AMY2)
At1g77380	amino acid permease 3 (AAP3)
At2g02020	NRT1/ PTR FAMILY 8.4
At2g02130	low-molecular-weight cysteine-rich 68 (LCR68)
At2g04160	Subtilisin-like serine endopeptidase family protein (AIR3)
At2g19590	ACC oxidase 1 (ACO1)
At2g22860	phytosulfokine 2 precursor (PSK2)
At2g30070	potassium transporter 1 (KT1)
At2g37130	Peroxidase superfamily protein
At2g44380	Cysteine/Histidine-rich C1 domain family protein
At2g46690	SMALL AUXIN UPREGULATED RNA 32 (SAUR32)
At3g09260	BGLU23
At3g12730	SAPL
At3g12750	zinc transporter 1 precursor (ZIP1)
At3g14560	hypothetical protein
At3g14840	LYSM RLK1-INTERACTING KINASE 1 (LIK1)
At3g15950	DNA topoisomerase-related
At3g16450	JACALIN-RELATED LECTIN 33 (JAL33)
At3g16460	JACALIN-RELATED LECTIN 34 (JAL34)
At3g20370	TRAF-like family protein
At3g21770	Peroxidase superfamily protein
At3g23050	indole-3-acetic acid 7 (IAA7)
At3g60720	plasmodesmata-located protein 8 (PDLP8)
At3g63110	isopentenyltransferase 3 (IPT3)
At4g12470	azelaic acid induced 1 (AZI1)
At4g12550	Auxin-Induced in Root cultures 1 (AIR1)
At4g14465	AT-hook motif nuclear-localized protein 20 (AHL20)
At4g15660	GRXS8

At4g15690	GRXS5
At4g19840	phloem protein 2-A1 (PP2-A1)
At4g21960	Peroxidase superfamily protein
At4g27410	NAC (No Apical Meristem) domain transcriptional regulator superfamily protein (RD26)
At4g32290	Core-2/I-branching beta-1,6-N-acetylglucosaminyltransferase family protein
At4g32870	Polyketide cyclase/dehydrase and lipid transport superfamily protein
At4g35480	RING-H2 finger A3B (RHA3B)
At4g36410	ubiquitin-conjugating enzyme 17 (UBC17)
At4g37540	LOB domain-containing protein 39 (LBD39)
At5g01210	HXXXD-type acyl-transferase family protein
At5g01840	ovate family protein 1 (OFP1)
At5g02260	expansin A9 (EXPA9)
At5g02600	NaKR1
At5g07010	sulfotransferase 2A (ST2A)
At5g18240	myb-related protein 1 (MYR1)
At5g23820	MD2-RELATED LIPID RECOGNITION 3 (ML3)
At5g24800	BASIC LEUCINE ZIPPER 9 (BZIP9)
At5g26260	TRAF-like family protein
At5g26280	TRAF-like family protein
At5g28770	BASIC LEUCINE ZIPPER 63 (BZIP63)
At5g43380	type one serine/threonine protein phosphatase 6 (TOPP6)
At5g43580	UNUSUAL SERINE PROTEASE INHIBITOR (UPI)
At5g49660	CEPR1 / XIP1
At5g54130	Calcium-binding endonuclease/exonuclease/phosphatase family
At5g59080	hypothetical protein
At5g63710	Leucine-rich repeat protein kinase family protein
At5g64120	Peroxidase superfamily protein
At5g65970	Seven transmembrane MLO family protein (MLO10)

Supplementary Table 2

Primers used in this study for qRT-PCR

name	sequence (5'-3')
CPD-L	AACCCTTGGAGATGGCAGA
CPD-R	GTAACCGGGACATAGCCTTG
DWF4-L	TTCTCGTTATGACCAACCTAATCTC
DWF4-R	AGGATGACGCTCCGTTGTT
UBQ14-L	TCCGGATCAGCAGAGGTT
UBQ14-R	TCTGGATGTTGTAGTCAGCAAGA
APL-L	TGGATATTCAGCGCAACGTA
APL-R	TGCACTTCCATTTGCATCTC
SUC2-L	TAGCCATTGTCGTCCCTCA
SUC2-R	CCACCACCGAATAGTTCGTC
IRX3-L	TGACATGAATGGTGACGTAGC
IRX3-R	CATCAAATGCTCCTTATCACCTT
SEOR1-L	AAGACACCAACGCCTCCA
SEOR1-R	CGATAGCATAGGAGACACTATCAAGA
CALS7-L	GCAGTAATGGAACTCCCTGAGA
CALS7-R	GGCTGAATGGAATCTTGGTC
SAPL-L	AGAGCCATCTCCAGAAGTTCA
SAPL-R	CCTTCGAAGATCCAACATGG
TCH4-L	GCTCAACAAAGGATGAGATGG
TCH4-R	CCTCTTCGCATCCGTACAAT
BR6ox2-L	CCCATGGAGATGGATGGA
BR6ox2-R	CTTTCCAGGGCAAAGCCTA
SULTR2:1-L	AACGATCTCATGGCTGGTTTA
SULTR2:1-R	TTGCATAACCAATGCTCTGC
NaKR1-L	GCTCAGTTTTGGCCTGAGATT
NaKR1-R	GTGGTGAATCAGCCAGTCCT
CEPR1-L	TATGGCTGGCACCTATGGTT
CEPR1R	GATCGTTGCTTTGGACGAGT
FTIP1-L	GCGCAAGATGTTGAGCCTA
FTIP1-R	TTGTACTTTAACGAAAGCTTGAGG
MYR1-L	GAAGTAGACGAAAGTCACAGTGAGAG
MYR1-R	GGCATCACTTATGGGTAAGTTCA
