# Supplementary figures and images for: VISUAL-CC system uncovers the role of GSK3 as an orchestrator of vascular cell type ratio in plants
Source: Commun Biol. 2020 Apr 22;3:184. doi: 10.1038/s42003-020-0907-3 (PMC7176705; doi:10.1038/s42003-020-0907-3)

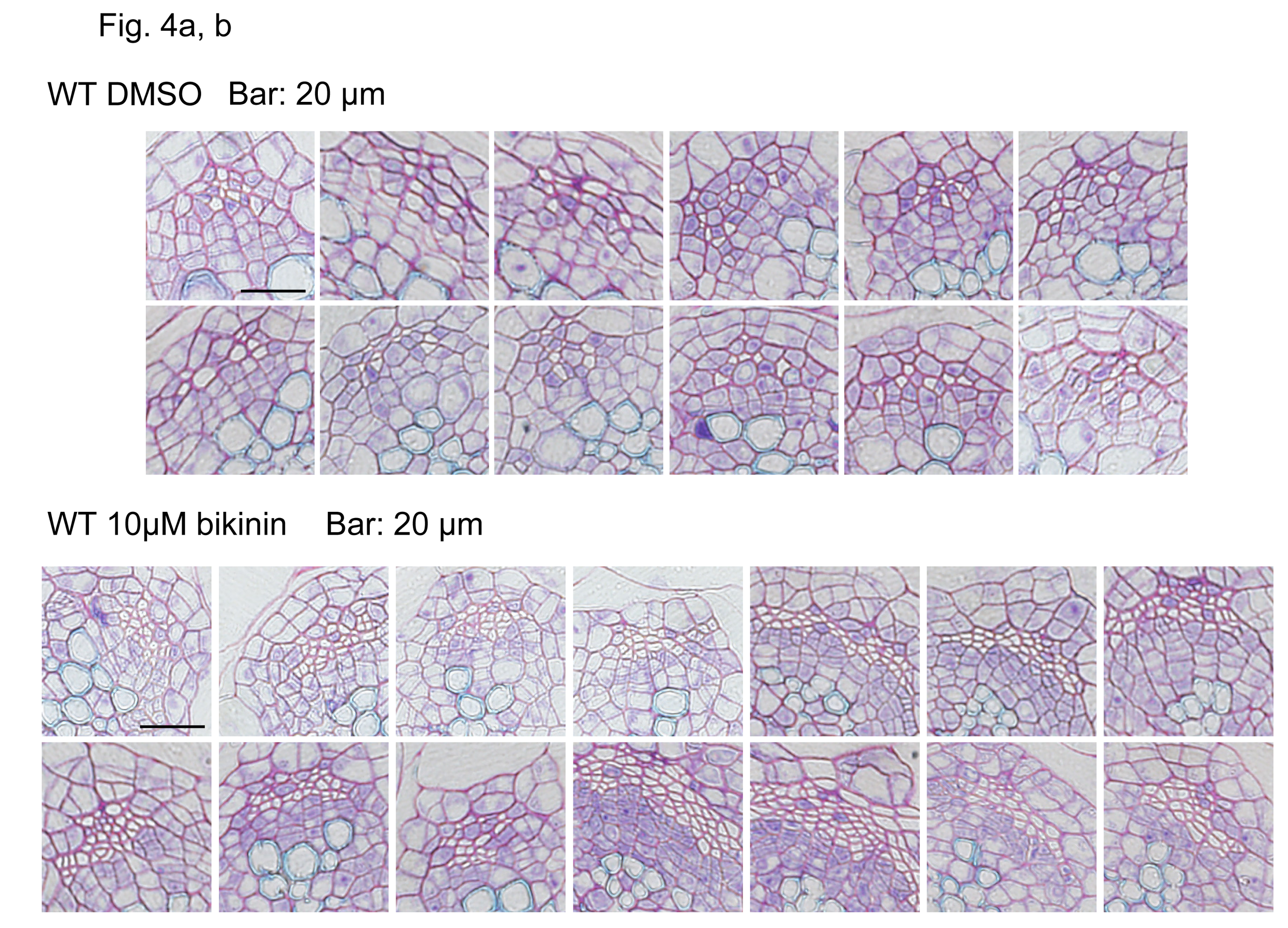

Supplement: Supplementary file 2 — Supplementary Data 1 [file 42003_2020_907_MOESM2_ESM.zip › Source data/source1.tif]

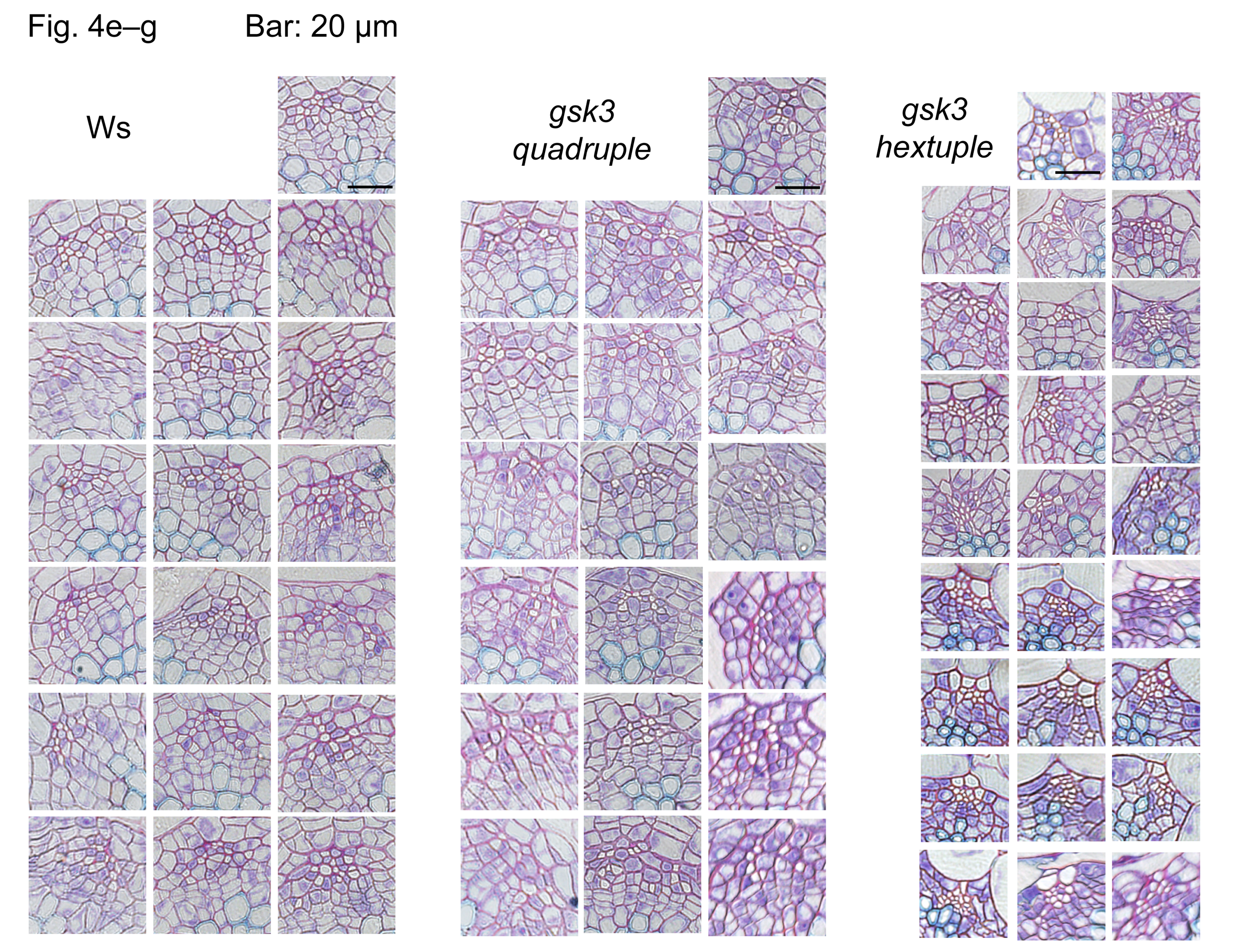

Supplement: Supplementary file 2 — Supplementary Data 1 [file 42003_2020_907_MOESM2_ESM.zip › Source data/source2.tif]

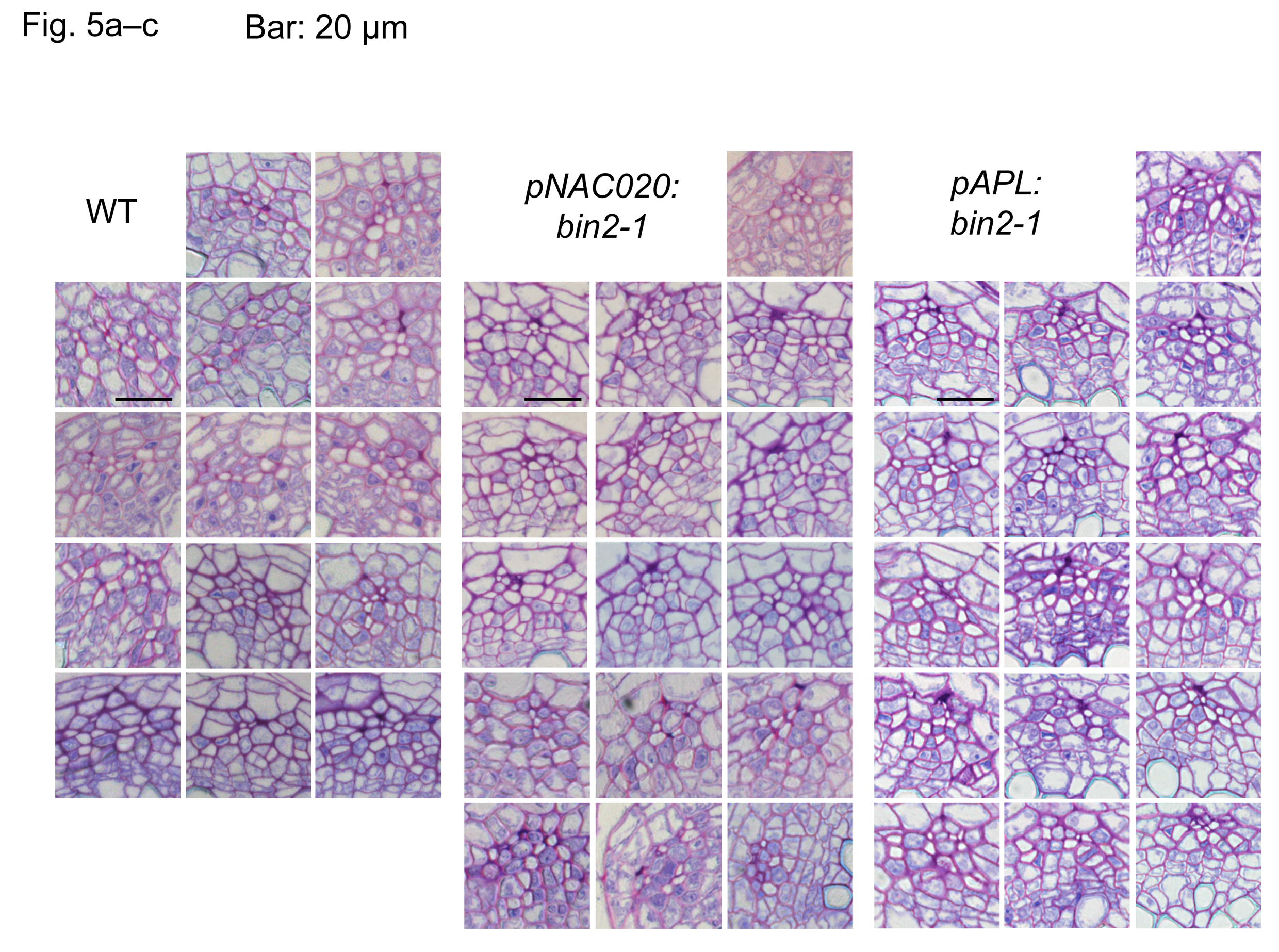

Supplement: Supplementary file 2 — Supplementary Data 1 [file 42003_2020_907_MOESM2_ESM.zip › Source data/source3.tif]

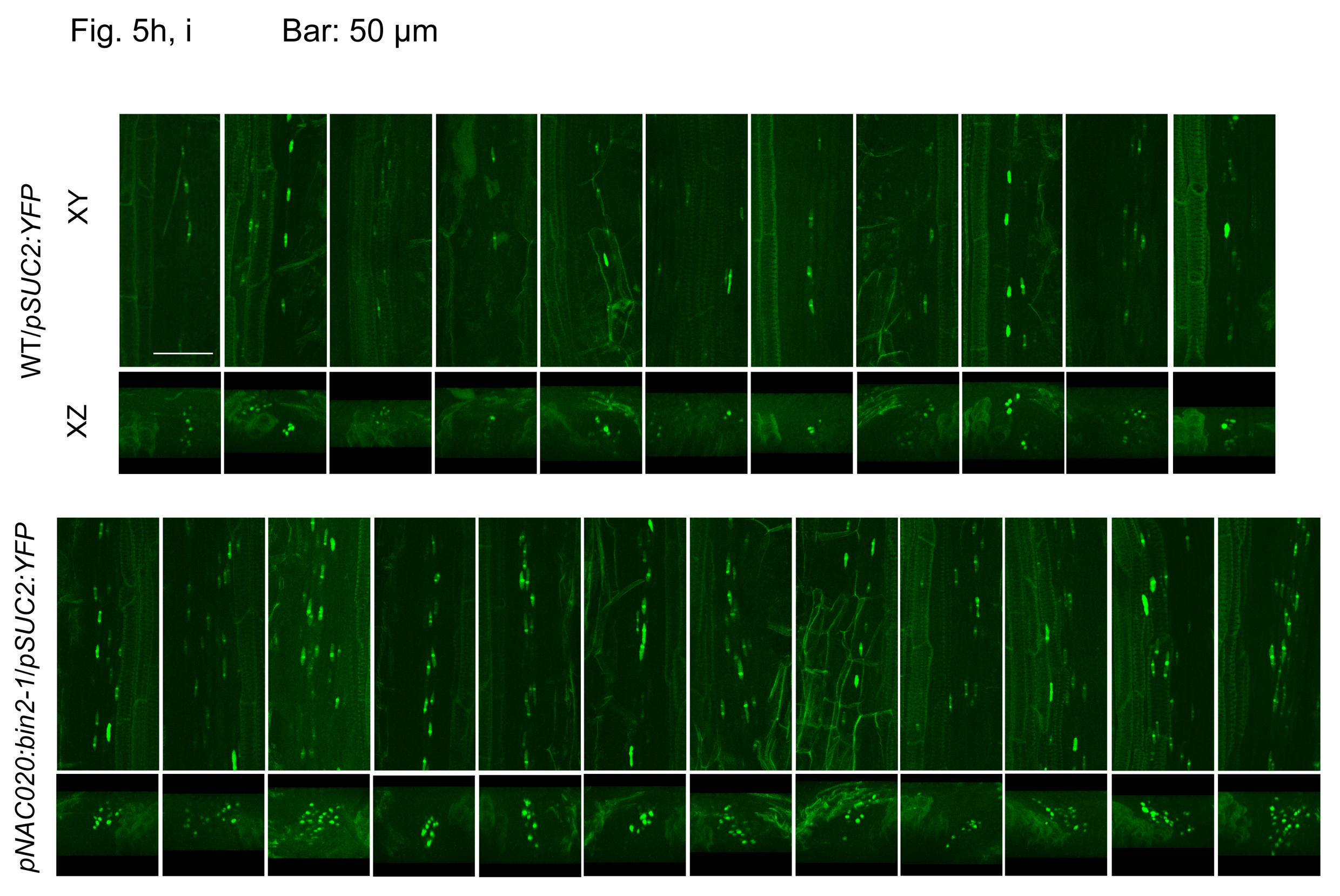

Supplement: Supplementary file 2 — Supplementary Data 1 [file 42003_2020_907_MOESM2_ESM.zip › Source data/source4.tif]
