# Supplementary material for: VISUAL-CC system uncovers the role of GSK3 as an orchestrator of vascular cell type ratio in plants
Source: Commun Biol. 2020 Apr 22;3:184. doi: 10.1038/s42003-020-0907-3 (PMC7176705; doi:10.1038/s42003-020-0907-3)
Supplement: Supplementary file 3 — Description of Additional Supplementary Files [file 42003_2020_907_MOESM3_ESM.pdf]

Description of Additional Supplementary Files

File Name: **Supplementary Data 1**

Description: **Source 1: All section images for Fig. 4a, b**
Source 2: All section images for Fig. 4e-g
Source 3: All section images for Fig. 5a-c
Source 4: All confocal section images for Fig. 5h, i
