# Supplementary material for: VISUAL-CC system uncovers the role of GSK3 as an orchestrator of vascular cell type ratio in plants
Source: Commun Biol. 2020 Apr 22;3:184. doi: 10.1038/s42003-020-0907-3 (PMC7176705; doi:10.1038/s42003-020-0907-3)
Supplement: Supplementary file 5 — Peer Review File [file 42003_2020_907_MOESM5_ESM.pdf]

Editorial Note: *This manuscript has been previously reviewed at another Nature Research journal. The manuscript was considered suitable for publication without further review at Communications Biology*
